# A network approach to elucidate and prioritize microbial dark matter in microbial communities

**DOI:** 10.1038/s41396-020-00777-x

**Published:** 2020-09-22

**Authors:** Tatyana Zamkovaya, Jamie S. Foster, Valérie de Crécy-Lagard, Ana Conesa

**Affiliations:** 1 Department of Microbiology and Cell Science, Institute for Food and Agricultural Research, University of Florida, Gainesville, FL 32608 USA; 2 Department of Microbiology and Cell Science, Space Life Sciences Lab, Merritt Island, FL 32953 USA; 3 Genetics Institute, University of Florida, Gainesville, FL 32608 USA

**Keywords:** Microbial ecology, Metagenomics

## Abstract

Microbes compose most of the biomass on the planet, yet the majority of taxa remain uncharacterized. These unknown microbes, often referred to as “microbial dark matter,” represent a major challenge for biology. To understand the ecological contributions of these Unknown taxa, it is essential to first understand the relationship between unknown species, neighboring microbes, and their respective environment. Here, we establish a method to study the ecological significance of “microbial dark matter” by building microbial co-occurrence networks from publicly available 16S rRNA gene sequencing data of four extreme aquatic habitats. For each environment, we constructed networks including and excluding unknown organisms at multiple taxonomic levels and used network centrality measures to quantitatively compare networks. When the Unknown taxa were excluded from the networks, a significant reduction in degree and betweenness was observed for all environments. Strikingly, Unknown taxa occurred as top hubs in all environments, suggesting that “microbial dark matter” play necessary ecological roles within their respective communities. In addition, novel adaptation-related genes were detected after using 16S rRNA gene sequences from top-scoring hub taxa as probes to blast metagenome databases. This work demonstrates the broad applicability of network metrics to identify and prioritize key Unknown taxa and improve understanding of ecosystem structure across diverse habitats.

## Introduction

For billions of years, microbes and their metabolic activities have been shaping Earth’s physical, chemical, and mineralogical landscape. Although microbes comprise the majority of the planet’s biomass, most microbial species and their genomes remain uncharacterized [1, 2]. These unknown aspects of microbial life, colloquially called “microbial dark matter” [1, 3] represent a fundamental impediment to microbial ecology, as microbe-dominated ecosystems cannot be reliably characterized without a thorough understanding of the roles microbes and their gene products play in ecosystem processes.

Currently, most of our knowledge of the microbial world is skewed by a few taxa that lend themselves to cultivation and genetic manipulation. Of the cultivated species, 88% are derived from just four phyla *Proteobacteria, Firmicutes, Actinobacteria*, and *Bacteroidetes* [1]. The uncultured and unsequenced microbial majority on Earth likely represents major evolutionary lines of descent within the tree of life and is expected to have played key roles in ecosystem formation, evolution, and function. Without a mechanistic approach to characterize the roles of these currently Unknown taxa in the ecosystem, we will not have a full understanding of how these organisms impact their neighbors, environment, or life as a whole.

Recent efforts to provide insight into the uncharacterized and uncultured majority through next-generation sequencing technologies have significantly expanded the microbial tree of life [4–6]. Yet, despite the recent explosion of nucleic acid sequencing of microbial environments, much remains to be learned [7, 8]. Truly understanding the ecological roles of Unknown taxa within communities requires a more comprehensive assessment of why Unknown taxa persist, or with whom they interact on a global scale. More importantly, it is unclear whether the presence of these unknown organisms confers a value that is not already provided by other, well-characterized microbes within the same ecosystem.

To more fully understand microbial life, particularly the contributions of Unknown taxa, it is first critical to understand the connectivity and structure of microbial ecosystems. Networks have long been used as analytical tools to better understand species’ roles and interactions [9–14]. By mathematically modeling a microbial community as a network, where nodes are different species and edges represent their relationships [9, 15], researchers can depict species interactions and study the structure of the environmental system. Network metrics, such as hub score, betweenness centrality, closeness centrality, and degree centrality [9, 16, 17], can be used to quantitatively describe these communities and pinpoint the most important taxa of a given environment, thereby providing essential clues about how specific taxa or gene products may contribute to ecosystem functioning [18]. Degree centrality is the number of edges in a network that connects one node (in our case operational taxonomic unit (OTU) or taxon) and is a measure of the level at which an OTU co-occurs, i.e., is present in the same samples and at similar levels, with other OTUs [19]. Betweenness centrality measures the extent to which a node lies on paths between other nodes and can be used to identify which OTUs communicate most with other members of the community network, thereby revealing which taxa are necessary for the co-occurrence with nearby taxa [19, 20]. Closeness centrality measures how far a node is to all other nodes and can be used to find the most central taxa of a given community network [9]. Finally, members of a network that have both high degree and betweenness centrality are typically the most connected taxa within the community and are considered “hubs.” Hubs may have ecological relevance to the community as their removal affects the largest number of connections and paths, causing the highest impact on the connectivity of the network [19, 21]. Although many advances have been made in microbial ecology using network-driven approaches [22–37] few, if any, revolve around Unknown taxa. Most network analyses focus on the role of known species, usually excluding any taxonomically unassigned or ambiguous taxa in early filtering steps. If included, any unassigned taxa are only briefly mentioned in connection to their interaction with more characterized, abundant phyla, leaving the role of unknown and uncultured taxa largely unexplored.

Here, we present a network-based approach to assess the ecological relevance of Unknown taxa within a targeted community. To provide both a broad and accurate interpretation of results, a comprehensive dataset encompassing four different aquatic environments was compiled. Networks were constructed with and without the Unknown taxa and changes in the network metrics degree, betweenness, closeness, and hub score were evaluated. In this manner, the contribution Unknown taxa provide to the overall community structure was systematically evaluated and compared across taxonomic levels. To identify the most ecologically prominent components in each environment, the hub score of all nodes was calculated, and the frequency of Unknowns among the top hubs was noted. These hubs were then subsequently removed from the networks to assess their fragmentation. Thus, taxa with the highest hub scores were considered ecologically relevant and critical actors of their networks by their high connectedness and presence. These hub network analyses serve as signatures of the potential importance of the Unknown taxa and provide a means to prioritize Unknown hubs for future characterization efforts. We demonstrate one of several possible applications of this approach, using particularly relevant hub Unknowns as probes to detect novel adaptation-related genes within metagenome scaffolds. The application of network theory to identify and prioritize key unknown microbial members may thereby help shed light on potential adaptation mechanisms of successful Unknown taxa while enabling a more comprehensive understanding of ecosystem structure under a diverse range of environmental conditions.

## Results

### Overall strategy to detect the relevance of Unknown taxa

**Fig. 1:**
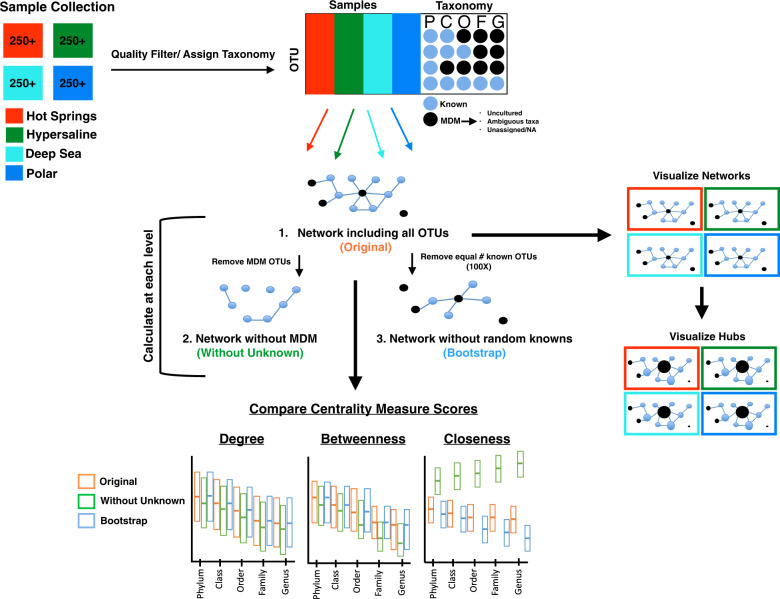
Overview of the analysis pipeline. A minimum of 250 samples was retrieved for each of the four different extreme environments—hot springs (red), hypersaline (dark green), deep sea (turquoise), and polar (blue). Sequence reads were quality filtered, assigned to a taxonomy, and clustered to OTUs. At each classification level, any unassigned, ambiguous, or uncultured OTUs were designated as Unknowns, or “microbial dark matter” (MDM). For each environment, at each classification level, the direct co-occurrence relationship between all OTUs was mathematically modeled as a network. Networks were created for each environment, across all taxonomic classification levels, including all OTUs (Original, orange), excluding MDM (Without Unknowns, light green), and excluding an equal number of random Knowns (Bootstrap, blue). Network centrality metrics (i.e., degree, betweenness, and closeness) were calculated for each node, compared, and visualized as boxplots between these network types. Hub scores were calculated for each node in the Original network and networks were recreated, resizing by hub score, where the largest size node indicates a top hub species.

**Fig. 2:**
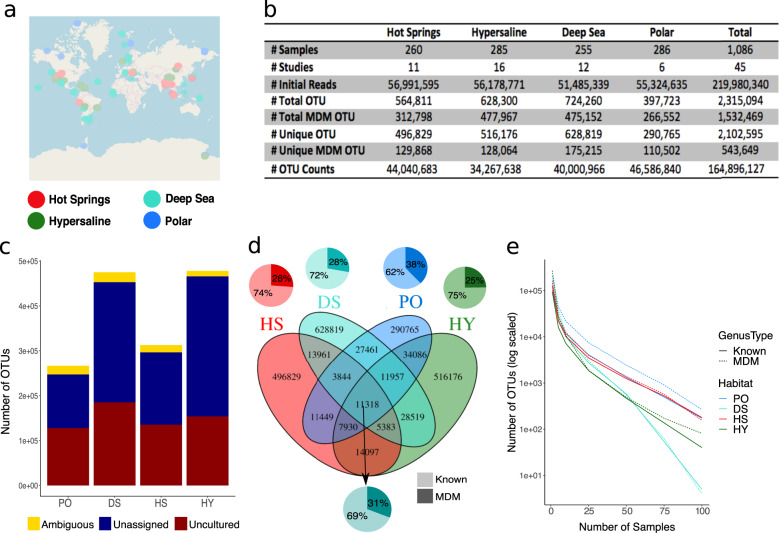
Summary of environmental 16S rRNA gene data. **a** Map of the sample sites used in this study. Circles symbolize sample locations and are color-coded by environment: hot springs (HS, red), hypersaline (HY, dark green), deep sea (DS, turquoise), and polar (PO, blue). **b** Summary of data used in this study. OTUs counts are provided at the genus level. **c** Proportion of “microbial dark matter” (MDM) OTUs for each environment labeled as unassigned (dark blue), uncultured (dark red), and ambiguous (yellow) after SILVA and UCLUST-based taxonomic assignment to OTU. **d** Venn diagram of shared OTUs in four extreme environments. Each pie chart depicts the proportion of unique OTUs that were Known (lighter shade) and Unknown (darker shade) for each environment, with the bottom-most pie chart showing combined data for all environments. **e** Prevalence curves indicate the number of unique OTUs consistently present at an increasing number of samples. Dotted lines signify the prevalence of MDM OTUs and solid lines signify the prevalence of Known OTUs.

A pipeline based on network analysis was developed to detect and quantitatively measure the overall and individual impact of Unknown taxa on their environmental communities (Fig. 1). Briefly, Illumina 16S rRNA sequencing fastq files belonging to four distinctive aquatic extreme environments (i.e., hot springs, hypersaline, deep sea, and polar habitats that included both Arctic and Antarctic samples) were collected from public databases and 45 different BioProjects (Fig. 2a and Supplementary Dataset S1). We included different environment types to assess general and environment-specific patterns and chose to use extreme habitats as they comprise some of the harshest and relatively understudied habitats on Earth, and therefore, are likely to contain uncharacterized organisms.

After quality filtering, reads were mapped to OTUs and annotated against the SILVA (v128) reference database [38] by an open-reference strategy, i.e., allowing the detection of Unknown OTUs [39]. Over two million Known and novel taxa were observed with the vast majority of the taxa annotated as Bacteria. Only the bacterial taxa or OTUs unclassified at the domain-level were targeted for downstream network analyses to demonstrate the feasibility of this approach across ecosystems. As the term “microbial dark matter” can have a broad meaning, here, we define Unknown taxa as uncultured, unassigned, or ambiguous by the reference database at each taxonomic classification level (e.g., phylum to genus). For each environment, networks reflecting across-samples co-occurrence relationships between all taxa, Known and Unknown, were constructed and referred to as the “Original” networks (Fig. 1). To assess the role of the Unknown taxa on network structure and properties, the Unknown nodes were removed from the ‘Original’ network and a new network, referred to as ‘Without Unknown’ was reconstructed. To ensure that changes in network properties were not caused just by the number of nodes, a third network, referred to as the “Bootstrap” network, was created where a random set of nodes of the same number as the Unknown OTUs was removed. The relevance of the Unknown taxa was assessed by comparing changes in degree, closeness, and betweenness scores between the three network types and by evaluating the frequency of Unknowns as top hubs within each of the “Original” environmental networks.

### A similar and significant fraction of Unknown taxa populates diverse environments

To assess whether there were distinctive patterns or trends of Unknown taxa within the four targeted environments, data from each habitat type were mined from several geographical locations across the globe (Fig. 2a and Supplementary Dataset S1). Reads were collected from the online repositories National Center for Biotechnology Information Sequence Read Archive (NCBI SRA) and Joint Genome Institute Genomes Online Database (JGI Gold). For each environment, between 255 and 286 16S rRNA samples and between 51 and 57 million reads were included in the analysis, resulting in a total of 219,980,340 reads from 1086 samples (Fig. 2b).

After processing, quality filtering, and OTU assignment steps were completed, there were 2102,595 unique OTUs totaling 164,896,127 amplicon read counts derived from these samples (Fig. 2b). The relative proportions of Unknown OTUs, which were designated as unassigned, ambiguous, or uncultured by the SILVA database, were compared between environments. Results indicated that all environments showed similar relative contributions of these three Unknown types, with unassigned and uncultured OTUs making up the majority of the Unknown component composition (Fig. 2c). Regardless of environment type, within each of the four habitats, between 25 and 38% of unique OTUs were cataloged as Unknown (Fig. 2b, d). Samples collected from polar habitats were significantly enriched (Fisher’s Exact Test *p* value < 0.05) in Unknown OTUs despite having the highest total read counts. The higher proportion of Unknown OTUs in the polar samples compared to the other habitats likely reflects the less-well-characterized biological diversity of these Arctic and Antarctic ecosystems.

Next, the proportion of shared Known and Unknown OTUs between environments was evaluated. Most OTUs, regardless of assigned or unassigned taxonomic status, were environment-specific, with only 11,318 out of the 2,102,595 OTUs present in all four of the environments (Fig. 2d). The majority of shared OTUs were observed between the hypersaline and polar environments and between hypersaline and hot springs environments, possibly reflecting the widespread distribution of hypersaline habitats across diverse thermal zones. Unsurprisingly, polar and hot springs environmental samples shared the least number of OTUs (Fig. 2d). Given this low common OTU pool across environments, network analyses were applied to each environment independently.

Last, the prevalence (i.e., the percentage of samples with nonzero counts in which an OTU was detected) of Known and Unknown OTUs was evaluated at the genus level to assess the consistency of OTU detection within each environment. The OTU matrix was sparse, with the majority of taxa observed in ≤50 (~20%) samples (Fig. 2e). However, prevalence curves were generally very similar for Known and Unknown OTUs in all four environments, indicating that Unknown OTUs are not necessarily rarer than already characterized species (Fig. 2e). Moreover, we confirmed that Unknown OTUs, like Known OTUs, were generally present and consistent across multiple studies within the same environment and did not tend to concentrate in any particular project (Supplementary Figs. S1–4). Consequently, these results indicated that a network analysis of these data would be a reflection of the co-occurrence structure of the community and not of potential compositional bias.

### Network analysis of OTU abundance at different taxonomic levels reveals the connectivity of unknown microbes

Having demonstrated that the Unknown taxa comprise a substantial proportion of unique OTUs and have comparable abundances to Known taxa within a community, network metrics were used to effectively compare the ecological relevance of both Known and Unknown taxa in subsequent networks. Microbial association networks were constructed that featured only significant co-occurrence correlation relationships for OTUs with a notable prevalence in each environment, meaning that any OTUs that were not detected in a sufficient number of samples were removed. To select a suitable prevalence threshold, the proportion of Known and Unknown taxa across a range of sample percentages was evaluated (Supplementary Fig. S5). Across all taxonomic levels, the Known and Unknown taxa of hot springs and polar habitats were more prevalent than those of hypersaline and deep sea communities; therefore, a slightly more stringent prevalence threshold (40%) was chosen for the former, and a lower threshold value (30%) chosen for the latter environments. These thresholds resulted in the retention of a similar fraction of data from the initial OTU count (Table 1), with 102–297 nodes present per environment, making the networks both suitably large and comparable.

**Table 1:** Breakdown of node and edge attributes across extreme environmental networks.

Environment	Prevalence threshold in number of samples	Number of selected nodes [fraction from initial data]	Number of connected nodes [number of Unknown at genus]	Number of edges in network [K-K, K-U, U-U at genus]	Ratio of number edges vs. number nodes
Hot Springs	104	291 [4.4E − 4]	290 [133]	552 [167, 230, 155]	1.9
Hypersaline	85	193 [3.7E − 4]	191 [118]	516 [89, 125, 302]	2.7
Deep Sea	75	102 [1.6E − 4]	97 [51]	194 [59, 65, 70]	1.9
Polar	113	279 [9.6E − 4]	274 [171]	797 [112, 244, 441]	2.9

K-K: the edge connects two Known nodes at the genus level.

K-U: the edge connects one Known and one Unknown node at the genus level.

U-U: the edge connects two Unknown nodes at the genus level.

The SpiecEasi Meinshausen–Buhlmann (MB) neighborhood algorithm was then used to construct networks (see “Methods”) that contained at least 100 nodes (i.e., OTUs) per environment and had edge-to-node ratios that varied from 1.9 and 2.9 (Table 1). Although most OTUs that passed the prevalence criteria became elements of the networks, no relationship between the initial data size (i.e., number of samples and taxa) and the interconnectedness (i.e., nodes/edges ratio) of the resulting network was observed (Table 1). The two environments with the highest number of initial OTUs (i.e., hypersaline and deep sea) had the lowest number of prevalent members, 193 and 102, respectively, and very different edge/node ratios, 2.7 and 1.9, respectively, indicating that high prevalence does not necessarily correlate with high co-occurrence. Similarly, the polar and hot springs networks retained a high number of prevalent OTUs but differed in edge number, yet again, indicating that the observed network structures were the result of the intrinsic properties of each environment and were not dependent on the sampling procedure. Interestingly, when evaluating the proportion of Unknown edges and Unknown-Unknown connections at the genus level, similar patterns were observed across environments. Between 45 and 62% of all connected nodes were Unknown OTUs and a higher proportion of Unknown-Unknown versus Known-Known links was present at the genus level (Table 1), for all environments but hot springs, where a higher proportion of Known-Unknown and Known-Known links was observed.

The results of this global analysis of network construction and composition demonstrate that although the general community connectivity might be environment-specific, the relative contribution of Known and Unknown taxa to these networks is similar. Once again, network properties were not a direct outcome of sampling biases, but more likely, reflect the biology of their respective ecosystems.

### Unknown taxa play important roles in interconnectedness and connectivity of extreme environmental microbial networks

**Fig. 3:**
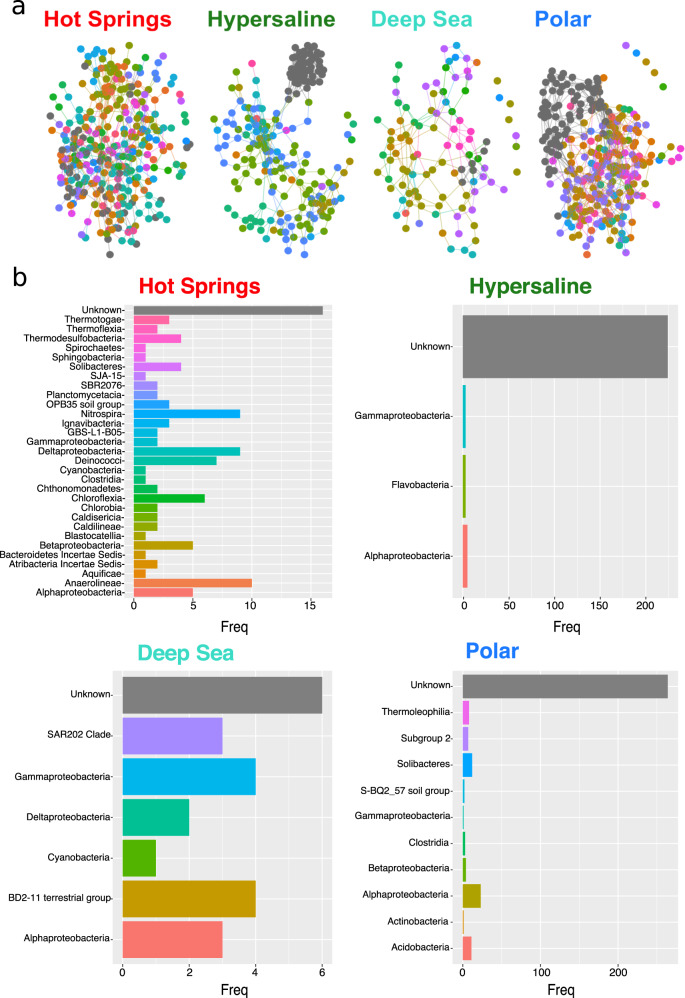
Analysis of environmental network taxa interconnectedness. **a** Microbial networks at class taxonomic classification level. Nodes are colored by class assignment, with gray nodes representing Unknown taxa at the class level. **b** Bar graphs of the co-occurrence relationships (i.e., edges) of Unknown OTUs with other taxa at the class level within each environmental network. *Y* axis labels and colors signify the different classes with which Unknowns were found to co-occur. Unknown-Unknown relationships are represented in gray.

Next, the position and neighborhood of Unknown nodes were examined. At the phylum level, Unknown taxa were present in the hot spring, hypersaline, and polar environments, but were not found in the deep sea network (Supplementary Fig. S6). The class level was the first taxonomic classification rank in which Unknown taxa were present in all environmental networks. To accurately assess the role of Unknowns, we evaluated class-level networks and observed that the hypersaline and polar Unknown OTUs created distinctive clusters, whereas hot springs and deep sea Unknown OTUs were intermixed with Known taxa (Fig. 3a). Hypersaline and polar Unknowns consistently appeared to be isolated and peripherally located compared to the centrally positioned hot springs and deep sea Unknown nodes across almost all classification ranks (Supplementary Fig. S6). Consequently, these results suggest that the clustering pattern is unrelated to a higher abundance of Unknowns and is more environment dependent. For example, the targeted hot spring environments had the highest number of Unknown OTUs yet showed the most dispersed connections between the these taxa (Fig. 3a). Thus, the inclusion of the Unknown taxa in our environmental networks models was, as anticipated, not solely the consequence of their level of prevalence, but rather a reflection of a particular abundance co-variation pattern.

For all four environments, Unknown OTUs had more frequently shared edges among them than with classified taxa (Fig. 3b). Unknown OTUs were found to frequently co-occur with each other within each environmental network, although the frequency of within-class interactions for Unknowns at the class level was found to be statistically no greater than all other within-class interactions for each environment (Supplementary Fig. S7). In fact, the pattern of a high frequency of shared edges among members of the same class held true for known classes as well (Supplementary Fig. S8). In accordance with other studies, these results demonstrate that OTUs of the same taxonomic classification most frequently co-occur with each other [40, 41]. Furthermore, the high frequency of shared edges between Unknown classes suggests that Unknown OTUs might be taxonomically related.

To ensure that the observations found were reproducible, robust, and not biased by earlier steps of the analysis, the diversity and position of Unknown taxa in the networks were examined for several parameters. Although the number of Unknown nodes changed at each level (Supplementary Fig. S6), the environment-specific network patterns observed at the class level (Fig. 3a) were retained at other taxonomic levels. In addition, to determine whether the topology of the network was a direct consequence of our correlation metric of choice or the prevalence threshold, three other network construction approaches were used: SparCC [11], CClasso [42], and Pearson correlation. Network analyses were performed across a range of prevalence thresholds (15–35%, at 5% increments). Again, regardless of the network construction approach or sample percentage applied, network shape and Unknown OTU position remained consistent and each environment exhibited a distinctive pattern of Unknown taxa inclusion. For example, Unknown nodes continued to occupy peripheral positions for hypersaline and polar networks, whereas nodes in hot springs and deep sea environments were more centrally located when applying different correlation metrics (Supplementary Fig. S9). Although networks appeared “noisy” at more lenient prevalence thresholds (15–20%; Supplementary Figs. S10–13), the networks and positioning of Unknowns at higher percent thresholds were similar in appearance to the “Original” networks for all environments. Based on these results, we found that our general analysis strategy was robust across parameter choices and, therefore, these networks captured critical features of the relationships among taxa within each distinctive environment.

### Microbial dark matter acts as unifiers and frequent hubs within extreme environmental networks

**Fig. 4:**
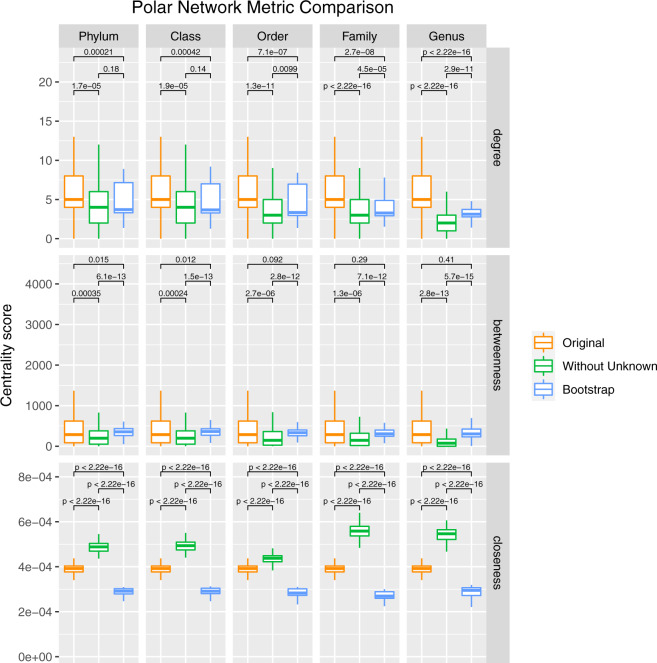
Impact of Unknown taxa on polar network metrics at different taxonomic levels. Boxplots of degree, betweenness, and closeness centrality values of nodes present in different network types at different taxonomic levels. Wilcoxon pairwise comparisons were used to assess significance between the three network types (Original-Without Unknown, Without Unknown-Bootstrap, Original-Bootstrap) for each taxonomic level. For each comparison, *p* values after Holm adjustment are shown.

Although these results suggest that Unknown taxa were highly interconnected, these observations did not reveal how the presence of Unknowns affected the overall community structure. To more fully understand this role, we analyzed how network properties changed in the presence and absence of Unknown OTU nodes. We evaluated changes in degree, betweenness, and closeness, as different network metrics reveal different aspects of the relevance of nodes within their networks. This approach has the potential to ascertain whether certain Unknown OTUs were more centrally positioned (e.g., due to high closeness scores), more essential for joining other taxa (e.g., high betweenness), or simply more prevalent and likely to co-occur with others (e.g., high degree). To control for the effect of node removal and distinguish effects of Unknown taxa from network size, networks were generated that excluded several randomly picked Known nodes equal to the number of Unknown OTUs. This process was repeated 100 times to create a distribution of “Null” or “Bootstrap” networks for statistical comparisons. Differences in network parameters between networks without Unknown OTUs and the “Original” or “Bootstrap” networks were determined by the Wilcoxon test and *p* values were adjusted using the Holm method [43]. Strikingly, removal of the Unknown taxa caused a statistically significant impact on all measured network metrics in all four studied environments (Table 2, Fig. 4, and Supplementary Figs. S14–24). In the polar environments, for example, removal of Unknown OTUs caused a significant decrease in degree (*p* value < 1E − 5) and betweenness (*p* value < 1E − 4) scores and a significant increase in closeness (*p* value < 2.22E − 16) scores across multiple taxonomic levels (Fig. 4), suggesting that the Unknown taxa in the polar environments are critical to the community structure and preserve local connections. Closeness score was the only parameter that behaved differently across environments (Table 2), increasing upon exclusion of the Unknowns for hypersaline and polar networks but decreasing at family level for the deep sea environment, due to the different topology of Unknown OTUs within their environmental networks. For example, the removal of centrally located Unknowns at the family level in the deep sea network increased node distance and network fragmentation. In contrast, in the hypersaline and polar networks, the removal of the peripherally located Unknowns resulted in a more connected network structure and appearance. The impact of Unknown node removal on degree, betweenness, and closeness persisted across taxonomical levels for the respective environments (Fig. 4 and Supplementary Figs. S14–16) and followed a similar pattern across tested correlation metrics (Supplementary Figs. S17–20) and prevalence thresholds (Supplementary Figs. S21–24).

**Table 2:** Impact on network metrics of the removal of Unknown OTUs from the network.

Environment	Degree	Betweenness	Closeness	Proportion of Unknown OTUs out of 20 top hubs
Hot Springs	↓ (F, G)	↓ (G)	n.s.	40%
Hypersaline	n.s.	↓ (G)	↑ (P to F)	100%
Deep Sea	↓ (all)	↓ (F, G)	↓ (F)	20%
Polar	↓ (all)	↓ (P to G)	↑ (P to G)	90%

Downward pointing arrow: significant decrease; upward pointing arrow: significant increase.

*P* Phylum, *F* Family, *G* Genus, *all* all taxonomic levels, *n.s.* not significant.

In summary, despite these minor differences in closeness score changes, these results suggest that Unknown OTUs had significant and comparable relevance for community structure in all four environments. The exclusion of Unknown nodes from their environmental networks led to a drastic change in network structure and interconnectedness, illustrating that the Unknown taxa are critical for establishing co-occurrence relationships and for maintaining overall network shape within each distinct ecosystem.

### Unknown taxa act as important hubs within extreme environment networks

After studying the overall relevance of the “microbial dark matter” in extreme environmental networks, we then concentrated on finding the most prominent Unknown components of each community. To do so, the hub score of each node was calculated to determine which taxa caused the most fragmentation or loss of network structure when removed, and therefore, may be a critical component for the microbial community structure of the target environment (Supplementary Dataset S2).

**Fig. 5:**
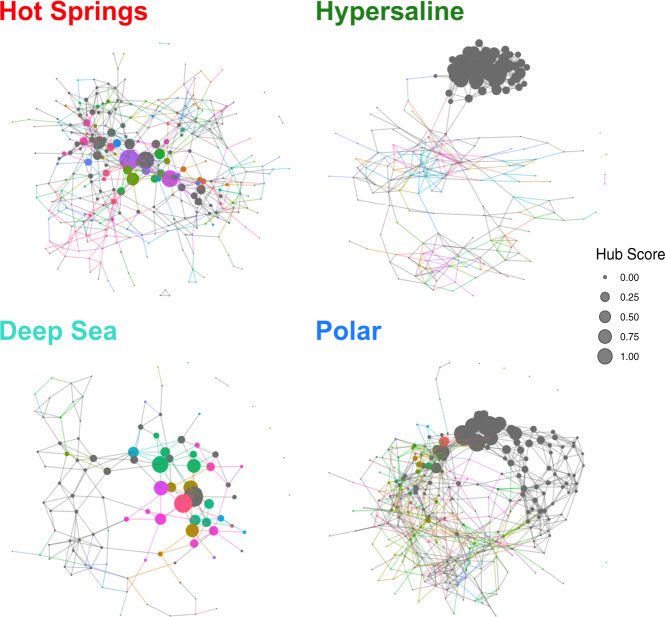
Hub analysis of extreme environmental networks. Environmental networks at the genus level with nodes sized as a function of hub score. Nodes are colored by genus classification with ambiguous, unassigned, or uncultured taxa depicted in dark gray.

Hub scores were calculated at the genus level for each node and the environmental networks were recreated, resizing the nodes as a function of the scores (Fig. 5). The overwhelming majority of the top hub scores, as symbolized by the largest-sized nodes in each network, were unknown for the hypersaline and polar environments, even at higher taxonomic ranks (Supplementary Fig. S25), different prevalence thresholds (Supplementary Figs. S26–29), or correlation metrics (Supplementary Fig. S30). Moreover, at least four of the nodes within the top 20 hub scores were unknown at the genus level for all environments (Supplementary Dataset S2), providing further evidence that Unknown taxa are key components of microbial community structure in all four extreme environments.

Student’s *t* tests were performed to evaluate the statistical significance of the differences between hub score values of Known and Unknown genera. Unknown genera in the hypersaline (*p* value = 2.01E − 13) and polar (*p* value = 2.41E − 9) habitats had significantly higher hub scores than Known genera, whereas in the deep sea environments the Unknown nodes were marginally significant (*p* value = 1.77E − 3). No significant differences were observed between the Known and Unknown nodes (*p* value = 0.6) in the studied hot springs environment. Based on these analyses, we concluded that while Unknowns OTUs occupied and dominated key positions within the hypersaline and polar networks, all environments harbored relevant Unknown hubs within their microbial communities.

### Network analysis of Unknown hubs as a tool to prioritize taxa for the search of novel genes with targeted functions

The high frequency of currently unknown microbes as top hubs within their habitats implies that these organisms have high prevalence and co-occurrence within their microbial communities, indicating that they have successfully adapted to survive in these harsh ecosystems. Therefore, we hypothesize that our network approach, particularly using the hub score to prioritize Unknowns, could serve as a critical foundation to study novel pathways and gene functions present in these yet-uncharacterized microbes. This hypothesis, however, poses two problems. First, this network-based approach relies on 16S rRNA sequencing data, which holds no additional functional information, and second, reference genomes for these uncultured organisms are lacking. To circumvent these challenges, we used the 16S rRNA sequences of the top Unknown hubs to probe large metagenomics databases, where extensive and diverse genome information is available on these microbial habitats, thereby facilitating the recovery of gene content associated with these unknown organisms. In parallel, we searched the literature for gene functions described to be associated with adaptation to our four studied extreme environments, resulting in a list of 86 “adaptation”-related terms that mostly contained metabolic and stress response processes (Supplementary Table S1). Once high-confidence matching scaffolds were found, putative novel genes with the targeted functions could be computationally identified by searching for genes of unknown function in the neighborhood of genes previously annotated with the list of targeted terms.

To explore this idea, a blastn search was performed on the 16S rRNA gene sequences of the top five Unknown and Known hubs at the genus level for each environment against 100 draft and permanently assembled metagenomes in the IMG/M database [44]. Hits with high similarity (i.e., >95%) and equivalent partial taxonomic classification in SILVA to the query hub 16S rRNA gene sequences were selected. To make sure that this search was not misled by incorrect 16S rRNA gene assembly in the metagenome scaffolds, we selected 70 such 16S rRNA genes obtained as hits of Known OTUs and performed a blastn search of these hits against the NCBI Microbial Genomes database. We obtained that the great majority (84%) of these 16S rRNA genes in scaffolds had >98% identity and between 97 and 100% sequence coverage with their corresponding genome sequence, while remaining 16S rRNA gene sequences belonged to species poorly represented in the NCBI database. Therefore, we concluded that 1S rRNA gene sequences in metagenome scaffolds could confidently be used in our analysis pipeline. Identified scaffolds were then filtered for a high (≥ 50) gene content and searched for operons containing gene descriptions matching any of the terms in our “adaptation” list, as well as genes labeled as “hypothetical proteins” or of “unknown function.” Hierarchical clustering of gene distance between pairs of genes was used to identify putative operons, with all results validated by checking gene neighborhood information for each scaffold using the IMG/M genome browsing and annotation platform.

Overall, metagenomic screening with the 16S rRNA gene sequences of the top Known and Unknown hubs returned numerous high-match, gene-rich scaffolds that varied by environment (Supplementary Table S2). The variation between habitats likely reflected the compositional differences of the metagenome database. Nevertheless, similar numbers of genes labeled as “adaptation,” “hypothetical genes,” and similar “putative adaptation-related” operons (see “Methods” for assignment criteria) were identified for all environments, regardless of hub type, with scaffolds consistently containing an average of three to four potential adaptation-related genes to extreme environments. Interestingly, the average number of hypothetical genes found per scaffold differed most between environments. Deep sea hubs consistently had the lowest average number of hypothetical protein genes per scaffold (12.8 for Unknown, 20.6 for Known), whereas polar, hypersaline, and hot springs scaffolds had similar, larger numbers of hypothetical genes (up to 35 for Known hypersaline hubs). These results suggest that a low total scaffold count does not prohibit detection of a high, equally abundant, number of hypothetical genes across environments or hub types. Importantly, across all habitats, an equivalent mean number of putative operons, where hypothetical and environment-relevant genes were found in close genomic proximity, were detected. Altogether, we recovered 6,734 un-annotated genes present in 535 putative operon structures potentially involved in the metabolic and stress responses to extreme environmental conditions (Supplementary Table S2).

**Fig. 6:**
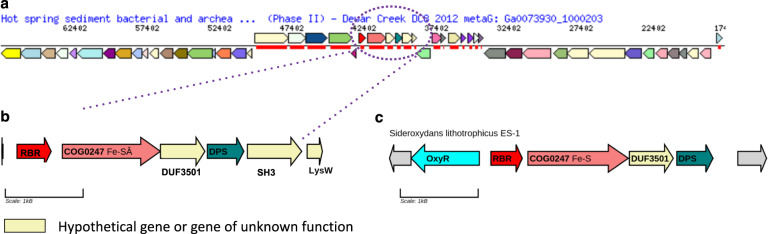
Metagenomics analysis of top Unknown OTU AB176701.1.1510. **a** Overview of operon annotation in scaffold hit Ga0073930_1000203 obtained from the JGI metagenomics browsing platform, which matched with 97% similarity to Unknown AB176701.1.1510. Genes in light yellow color represent hypothetical genes or genes of unknown function. **b** Zoom-in of the selected operon with adaptation-related gene annotations. Bioinformatic function predictions for genes of unknown function are indicated under their gene boxes. **c** DUF3501-RBR containing operon for *Sideroxydans lithotrophicus*, including the OxyR gene. RBR Rubrerythrin gene, COG0247 Fe-SA Fe–S oxidoreductase gene, DUF3501 domain of unknown function 3501, DPS DNA-binding ferritin-like protein, SH3 SH3-domain-containing protein, LysW lysine synthesis protein W. OxyR: hydrogen peroxide-inducible genes activator.

An example of the outcome of this approach for the targeted hot springs habitat is depicted in Fig. 6. Blast results showed a 97% sequence similarity match to the fasta sequence of Unknown hub AB176701.1.1510 (Supplementary Dataset S2) within the metagenomic scaffold Ga007390_1000203, which was originally generated from Dewer Creek hot spring sediment in British Columbia. The scaffold contained 98 genes in total, of which 19 were annotated as “Hypothetical Proteins” and distributed across seven operons (Fig. 6a). One of the identified operons contained three putative genes with predicted proteins of unknown function (i.e., two were labeled as hypothetical and one was labeled with unknown function). Further, this operon contained a gene annotated as Fe–S oxidoreductase and two well-known oxidative stress genes encoding DNA-binding ferritin-like protein (DPS) and rubrerythrin (RBR), suggesting possible roles in oxidative stress-related functions. An exact match for all six of the protein sequences in the same operonic structure was found in the genome of the bacterium *Blastocatellia* by searching the NCBI nonredundant database (Fig. 6b), which was recently sequenced as part of a hydrothermal vent metagenome project [45]. Further bioinformatic analysis of the poorly characterized open reading frames in this operon led to the identification of Ga0073930_100020343 as a domain of unknown function (DUF) DUF3501 family member and Ga0073930_100020344 as part of the COG0247 family (Fe–S oxidoreductase). The link with oxidative stress was reinforced by the clustering of these same four genes with the hydrogen peroxide-inducible genes activator OxyR in *Sideroxydans lithotrophicus* ES-1 (Fig. 6c). The two other hypothetical genes, Ga0073930_100020341 and Ga0073930_100020340, were identified as a putative SH3-domain-containing protein and a putative lysine synthase protein, respectively. Notably, the DUF3501-COG0247-RBR functional association had already been reported in a previous study where the authors proposed that the DUF3501 and COG0247 protein families enabled the functional adaptation of RBR from a thermophilic/anaerobic to a mesophilic/aerobic environment [46]. The identification of DUF3501 by our OTU prioritization analysis of the hot spring habitat supports this prior work suggesting the gene may be part of the microbial adaptation strategy to high temperatures. These results illustrate how the hub score prioritization can be successfully combined with computer-intensive mining of publicly available metagenomic data to identify novel and potentially ecologically relevant genes and gene products.

## Discussion

Microbial communities are complex and dynamic, however, with the vast majority of Earth’s microbes yet to be cultured or characterized, our understanding of these systems is likely limited or skewed by this large gap-in-knowledge. To more fully understand the impact of “microbial dark matter” on ecosystem structure and function, we have developed a network theory-based approach to assess the relevance of the uncultured and Unknown taxa within their microbial communities. Implementation of this bioinformatic pipeline demonstrated that: (1) specific patterns of the microbial network could be identified and compared for targeted ecosystems across taxonomic levels; (2) the comparison of centrality metrics between networks including and excluding the Unknown taxa is an effective strategy to reveal the relevance of these organisms within their communities, (3) Unknown taxa, just as Known taxa, can act as key hubs in ecosystem structure due to their high prevalence and strong central connections; and (4) network metrics can be used to prioritize Unknown taxa for downstream analysis by using the 16S rRNA gene sequences of top-ranked hubs as probes to screen publicly available metagenomic datasets for the identification of ecologically relevant gene functions.

### Harnessing the power of networks to elucidate “microbial dark matter”

No matter the environment, previous research has shown that “microbial dark matter” represents a significant limitation to the exploration of the global microbiome [1–3, 47–53]. To address and overcome this challenge, we developed a combined bioinformatics pipeline and network theory approach that was applied to a large, geographically diverse 16S rRNA dataset of four extreme aquatic environments to determine the ecological relevance of the unknown organisms in these communities. Although correlation-based microbial networks cannot infer the nature of ecological relationships, such as syntrophy or competition, they are indicative of social interactions within the community and can serve as important focal points for downstream analyses [54]. Our analysis clearly showed that the unclassified and uncultured taxa were prevalent and represented a significant proportion of the microbial diversity in all ecosystems examined, and therefore, should not be overlooked when examining community dynamics. Both Known and Unknown OTUs were found to be environment-specific, agreeing with previous reports of habitat-specific, niche-partitioning species of hypersaline lakes [55], deep sea vent communities [56, 57], and polar lakes [58, 59]. Furthermore, our work extended beyond simple composition analysis and demonstrated the consistent and significant contribution of Unknown OTUs to microbial community structure.

By using network metrics to study these four extreme environment networks, additional insights into the ecological relevance of these unknown organisms could be gained. The Unknown OTUs positively contributed to betweenness and degree centralities (i.e., denoting microbial interactions). More importantly, the exclusion of Unknown taxa was more detrimental to the overall network than the exclusion of random Known components, as more central node connections (e.g., ecotype interactions) were lost, causing a greater network fragmentation. Moreover, Unknown taxa established co-occurrence relationships with themselves, suggesting that they might be phylogenetically related, as is the case for Known taxa [40, 41]. The results presented here reveal that Unknown taxa are frequently key members of extremophilic microbial ecosystems and strongly advocate for the inclusion of Unknown taxa in any metagenomics or amplicon composition and interaction studies, as key biological interactions may remain undiscovered otherwise.

### Networks can prioritize the most ecologically relevant Unknown taxa in a community

Unclassified microbial taxa often occurred as top hubs across all examined environments. Since hubs, by definition, significantly contribute to network structure and cohesiveness, these unknown microbes can be considered keystone taxa, most likely playing vital and meaningful roles within key ecosystem processes in these habitats. Moreover, hub positions indicated that these Unknown taxa were prevalent in their environments and co-varied with many other Known taxa, and hence can be considered successful components of their microbial communities. During the elaboration of the network analyses described in this work, a new version (138) of the SILVA database was published. Re-running our pipeline with SILVA v138, and latest DADA2 (version 1.14.1)/DECIPHER (version 2.14.0) software [60, 61] returned similar results as presented here (not shown), confirming our conclusions held through software updates. These results support the value of our analysis and suggest that this approach could be used to identify the highly interacting OTU components of any microbial community. The frequent dominance of Unknown taxa as top-scoring hubs stresses the need for further exploration and functional characterization of these novel species and also offers new tools for prioritizing novel taxa for follow-up studies.

### Filling in gaps-in-knowledge of ecosystem functioning with hubs of Unknown taxa

Understanding the emergent properties of an ecosystem, i.e., those taxa, genes, and functions that are important for a particular niche, can have a big impact on our understanding of that environment. Using amplicon-based approaches to address these questions, however, can be limited. For example, amplicon-focused tools, such as PICRUSt [62] and Tax4Fun [63], can retroactively predict gene function from 16S rRNA gene data [64] under an assumption that taxonomy and function are well conserved. However, this approach might only work for major pathways and more importantly, these tools require reference genomes to be present and well-annotated for each ecotype, and therefore, cannot be applied to novel, uncultured organisms.

Here, we envisioned an alternative approach to probe metagenomics databases with 16S rRNA sequences prioritized from the top Unknown hubs of a given environment and used those sequences to investigate gene content of associated scaffold hits. While this is by no means a comprehensive characterization of the functional potential of unknown organisms in extreme environments, which would require a targeted experimental study, our approach leverages the wealth of metagenomics data currently available within public databases to gain novel insights on microbial function. These resources encompass hundreds of terabytes of data [7, 65] and represents untapped sources of valuable information that can, and should be, exploited both for fundamental science and for potential biotechnological applications.

The successful retrieval of unknown genes, potentially involved in environmental stress responses, from uncultured and unclassified organisms, indicated that this network and hub identification approach is an effective strategy to use prioritized OTUs for direct data-mining efforts. Notably, in this proof-of-concept effort, just a few top hub score OTUs within the hot spring networks were used to screen a fraction of the available metagenomics information, still recovering a substantial number of candidate genes. With one specific example, we illustrated the power of this methodology to unveil interesting gene functions. Supported by sufficient computational resources, up-scaling of this concept holds the potential for the large-scale discovery of novel gene functions and pathways, further unraveling roles that these Unknown taxa may play within their respective ecosystems.

In summary, this approach has the potential to be extended to other aspects of the environmental microbiome, including, but not limited to, the archaeal and eukaryotic taxa, as well as other multi-omic platforms (e.g., metaproteomics, metabolomics, and metatranscriptomics). As reference databases continue to grow, taxa and gene co-occurrence network analyses and measurements can also be used to evaluate changes in ecosystem structure over different temporal and spatial scales. The application of this strategy to a variety of microbial ecosystems from all environments could be used to more fully understand those features of the hidden microbial world that are critical for environment-specific or global attributes of microbial ecosystems.

## Methods

### Data retrieval

To mine samples from public databases, search queries of “16S,” “V4”, “V3,” “Illumina,” “hot springs,” “hypersaline,” “Arctic,” “Antarctic,” “deep sea,” and “hydrothermal” were utilized to find suitable studies from NCBI SRA and JGI Gold. A complete list of all samples and accession numbers for published data used in this study are listed in Supplementary Dataset 1. All samples were classified into their respective environmental categories (i.e., hot springs, hypersaline, deep sea, and polar) using available information provided by the studies in the public repositories. All raw data were converted to fastq format using the NCBI SRA Toolkit (http://ncbi.github.io/sra-tools/). Although the complete dataset included both paired-end and single-end samples, only the forward reads of paired-end samples were used in subsequent steps, due to the consistently noted lower quality of reverse reads of Illumina samples.

### Sample preprocessing, filtering, and OTU mapping

Quality filtering and preprocessing of all sequence data were performed through the split_librairies_fastq.py script from the Quantitative Insights into Microbial Ecology (QIIME) pipeline (Version 1.9.1) [39] using a Phred quality threshold of 19. All sequences passing quality filtering were clustered at 97% sequence similarity and classified to OTUs using the SILVA (v128) SSU reference database by the pick_open_reference_otus.py script. All singletons were discarded. The filter_taxa_from_otu_table.py script was used to remove any OTUs related to Archaea, mitochondria, or chloroplast so that the analysis would be targeted to the Bacteria. The collapse_samples.py script was used to compare OTU presence across environments (i.e., hot springs, hypersaline, deep sea, and polar). The filter_samples_from_otu_table.py script was used to separate the global OTU biom table into four environment-specific biom tables. All subsequent statistical and network analyses were conducted in R (v 3.5.1).

### Identification strategy for Unknown taxa

At each taxonomic level, from phylum to genus, Unknown taxa were identified as any OTU taxonomically assigned as: “uncultured,” “uncultured bacterium,” “Unknown,” “Unassigned,” “Ambiguous taxa,” or “NA” by the open-reference picking strategy. These OTUs were renamed as “Unknown” for all subsequent analyses and comparisons. An OTU was only labeled as “Unknown” at the specific taxonomic classification level at which it could not be taxonomically assigned beyond the higher classification descriptors. For example, if an OTU had a known order but unknown family description assigned by the reference database, it would only be designated as an Unknown in the network analysis for family and genus taxonomic classification and, at all higher classification ranks, would be referred to its known classification status.

### Network creation

To create networks of each environment, OTU biom tables and corresponding mapping information (i.e. sample ID, geographic location, longitude, and latitude) were imported into R using the package phyloseq (version 1.28.0) [66]. OTU tables, now converted into *phyloseq* objects, were filtered using the filterTaxonMatrix() function from phyloseq, keeping only taxa present in a given percentage (from 30–40%) of samples per environment to reduce sparsity and ensure robust results. For each environment, the filtered OTU *phyloseq* object was normalized, transformed, and converted into an adjacency matrix based on covariance by the spiec.easi() function from the package SpiecEasi (version 1.0.7) [67], using MB’s neighborhood selection (method = “mb” parameter) to estimate the conditional dependence of each pair of OTUs. This approach is robust and ideal for sparse, compositional amplicon, and metagenomic data and, unlike other correlation methods, prevents most spurious, indirect relationships from being included in the networks [67, 68]. The Stability Approach to Regularization Selection (StARS) method was used to find the optimal sparsity parameter, with the StARS variability (i.e., minimum *λ*) threshold set to 0.05 for all networks [69]. Each adjacency matrix was then converted into an *igraph* object and visualized as a network using the adj2igraph() and plot_network() functions from SpiecEasi. Networks were created and visualized at each taxonomic classification level, using the function plot_network() of phyloseq, with nodes representing OTUs and edges representing direct co-occurrence relationships between OTUs. Each resulting network contained at least 100 nodes (i.e., OTUs) per environment and edge-to-node ratios that varied from 1.9 to 2.9 (Table 1).

To create reduced networks, either excluding Unknown OTUs (“Without Unknown” network) or randomly selected Known OTUs (“Bootstrap” network), the delete_vertices() function (package igraph (version 1.2.5)) was used. To create bootstrap networks, Known nodes were randomly selected by the sample()function, with the x parameter equal to the total number of OTUs, Known and Unknown, at that specific taxonomic classification level for the chosen environmental network and size parameter equivalent to the number of Unknown OTUs for that network. Networks were created using the same SpiecEasi pipeline as described above. These randomly reduced networks were created 100 times for each taxonomic classification level from phylum to genus for each of the four target environments (i.e., hot springs, hypersaline, deep sea, and polar habitats).

### Neighborhood analysis

For each environmental network, the as_edgelist()function from igraph was applied to identify all edges between nodes. Using the taxonomic classification information found in the original OTU table that was retrieved by tax_table() function on the *phyloseq* object, all nodes were matched up with their taxonomic classification. A data frame containing all taxonomic information of each pair of connected OTUs was then used to identify which classes shared edges.

### Network analysis strategy

For each environment, at each taxonomic level from phylum to genus, network-level and node-level measures of networks including Unknown OTUs (i.e., “Original”), excluding them (i.e., “Without Unknown”), and excluding an equal number of randomly selected Known OTUs (i.e., “Bootstrap”) were evaluated and compared against each other and visualized as boxplots. The network measures evaluated for all nodes present within each network were degree, closeness, betweenness, and hub score and were calculated by using the igraph package functions: degree(), betweenness(), closeness(), and hub_score(). Wilcox test was used to evaluate the statistical significance of changes in degree, betweenness, and closeness between the three network types using the stat_compare_means()and compare_means() functions from the ggpubr (version 0.3.0) package. *P* values were adjusted using the Holm method [43], and boxplots were created using the package ggplot2 (version 3.3.0).

### Hub blast against metagenomes

For each environment, the fasta sequences of the five Known and Unknown taxa with the highest-ranked hub scores were retrieved with the *subseq* function from the SEQTK toolkit (version 1.0-r64-dirty) (https://github.com/lh3/seqtk), using a text file of the hub sequence names and the fasta file of complete sequences (*new_refseqs.fna*) produced by the *QIIME pick_open_reference_otus.py* script as the input. Next, using the selected genomes blast feature in IMG/M, we performed a *blastn* search of the five Known and Unknown hub sequences (using the default number of hits (500) and *e* value (1E − 05) specified) against 100 publicly available finished, draft, and permanent draft metagenomes pertaining to each environment. Blast results were exported and saved in.txt format and further analyzed in R. Only blast hits meeting ≥95% match and ≥95% query cover to the query hub sequence were retained. Of these hits, only metagenome scaffold hits, where at least 50 genes were present, were retained. For each scaffold that met the percent identity for the 16S rRNA gene (i.e., ≥95%) and gene number (≥50%) criteria, the gene content information was accessed manually and exported. The gene name, strand position, and start and end coordinate positions given in the file were used to identify the number of functionally annotated and unknown hypothetical protein genes and also used to identify putative operons of genes with similar functions. To verify that we were not being misled by misassembled 16S genes in the metagenome scaffolds, we obtained the 16S gene sequences from 70 scaffolds identified as hits of Known OTU hubs and conducted a blastn search against the Microbial Genomes database in NCBI. We evaluated the percent identity, taxonomic classification, and query cover results obtained after blast.

### Identification of hypothetical and putative adaptation genes and operons

The term “hypothetical” was used in the grep()function in base R to identify hypothetical protein genes on each strand for each scaffold. A list of keywords related to metabolic and extreme environmental stress response functions retrieved from literature was used to parse for adaptation gene matches among all genes present on each strand (Supplementary Table S1). To identify closely related genes, hierarchical clustering was performed based on the distance between gene start and end coordinate positions of each gene pair using the packages ape (version 5.3) and dendextend (version 1.13.4), and the function hclust()in base R. Closely clustered genes on a single strand (in the same direction) represented putative operons and any ten genes within 5000 bp or less to one another were considered to belong to one operon. If targeted genes were among the ten closest neighbors (i.e., <5000 bp away) to a hypothetical gene, we defined this set of hypothetical and potentially extreme stress adaptation-related neighboring genes as a putative adaptation operon. Gene clusters were then analyzed using the PubSeed database [70] and visualized with the Gene Graphics tool [71].

### Sample criteria validation

To account for the possible confounding effect of sample size, all networks were reconstructed and reanalyzed, using a range of different percentages (from 15 to 40% at 5% increments) of samples in the initial filtering process of the OTU table, discarding any taxa that did not meet this sample percentage threshold from being included in the networks. Networks excluding unknown and random Known nodes were constructed using the same methods as described previously and network measures were recalculated and visualized as boxplots to determine the effect of different sample size criteria and its statistical significance.

### Network tool validation

To determine whether other measurements of species’ co-occurrence relationships changed our final network analysis results, three other methods were used to calculate the relationship between pairs of species in R: SparCC [11], CCLasso [42], and Pearson correlation. The R rcorr()function, with the parameter type set to “pearson” from the package Hmisc (version 4.4-0) was used to calculate Pearson correlation. The R cclasso()function (https://github.com/huayingfang/CCLasso/blob/master/R/cclasso.R) was used to calculate CCLasso correlation, and the adapted sparcc()function in the SpiecEasi package was used to calculate SparCC correlation. Subsequent networks were created using igraph. Boxplots were created to compare the median network measure scores of degree, betweenness, and closeness for the three network types at all classification levels for the four (SpiecEasi, SparCC, CCLasso, and Pearson) correlation networks.

### Scripts and documentation

To encourage a deeper investigation into the role of microbial dark matter, ready-to-use scripts, and documentation to apply this methodology to other ecosystems are available. All scripts used in this analysis, along with a complete documentation of the bioinformatics pipeline, are available at http://github/Conesalab/MDM.

## Supplementary information

Supplementary Materials

Supplementary Dataset S1

Supplementary Dataset S2
