# Supplementary material for: A network approach to elucidate and prioritize microbial dark matter in microbial communities
Source: ISME J. 2020 Sep 22;15(1):228–44. doi: 10.1038/s41396-020-00777-x (PMC7852563; doi:10.1038/s41396-020-00777-x)
Supplement: Supplementary file 1 — Supplementary Materials [file 41396_2020_777_MOESM1_ESM.pdf]

Supplementary Materials

A network approach to elucidate the relevance of microbial dark matter in extreme aquatic environments

Tatyana Zamkovaya[1], Jamie S. Foster[2], Valérie de Crécy-Lagard[1,3], Ana Conesa[1,3*]

[1] Department of Microbiology and Cell Science, Institute for Food and Agricultural Research, University of Florida, Gainesville, FL 32608

[2]Department of Microbiology and Cell Science, Space Life Sciences Lab, Merritt Island, FL 32953

[3]Genetics Institute, University of Florida, Gainesville, FL 32608

Running title: Identifying hubs of microbial dark matter in extreme environments

*Corresponding author: aconesa@ufl.edu

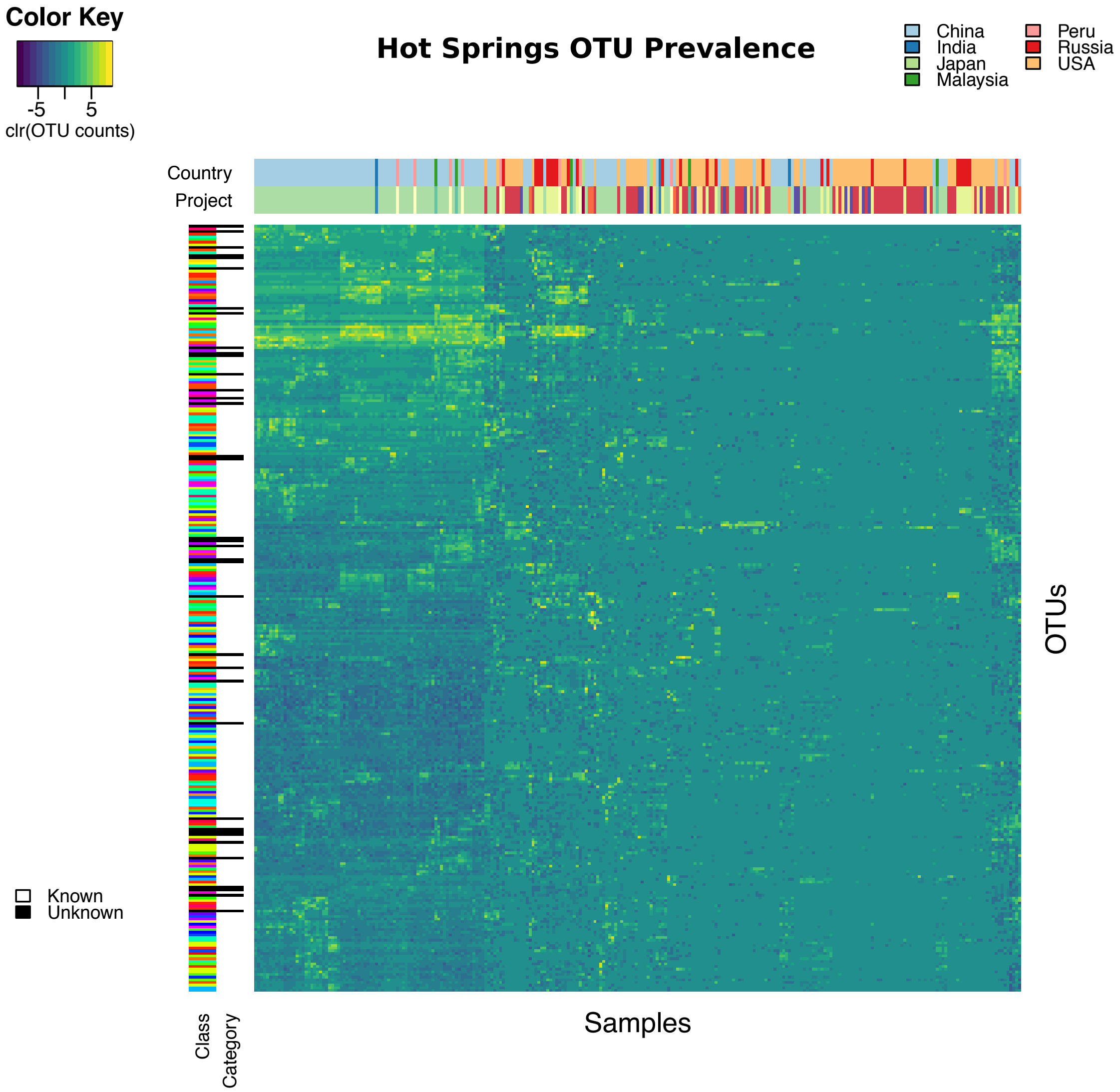

Supplementary Fig. S1. Taxa prevalence heatmap for hot springs network. Relatve abundance (i.e., OTU counts) after centered-log-ratio (clr)-normalization, across all samples, is visualized using a blue-yellow color-scale mapping, with blue signifying low prevalence and yellow signifying high prevalence. By row, color bars depict OTU class and category (Unknown, black; Known,white). By column, color bars indicate sample contry and project source.

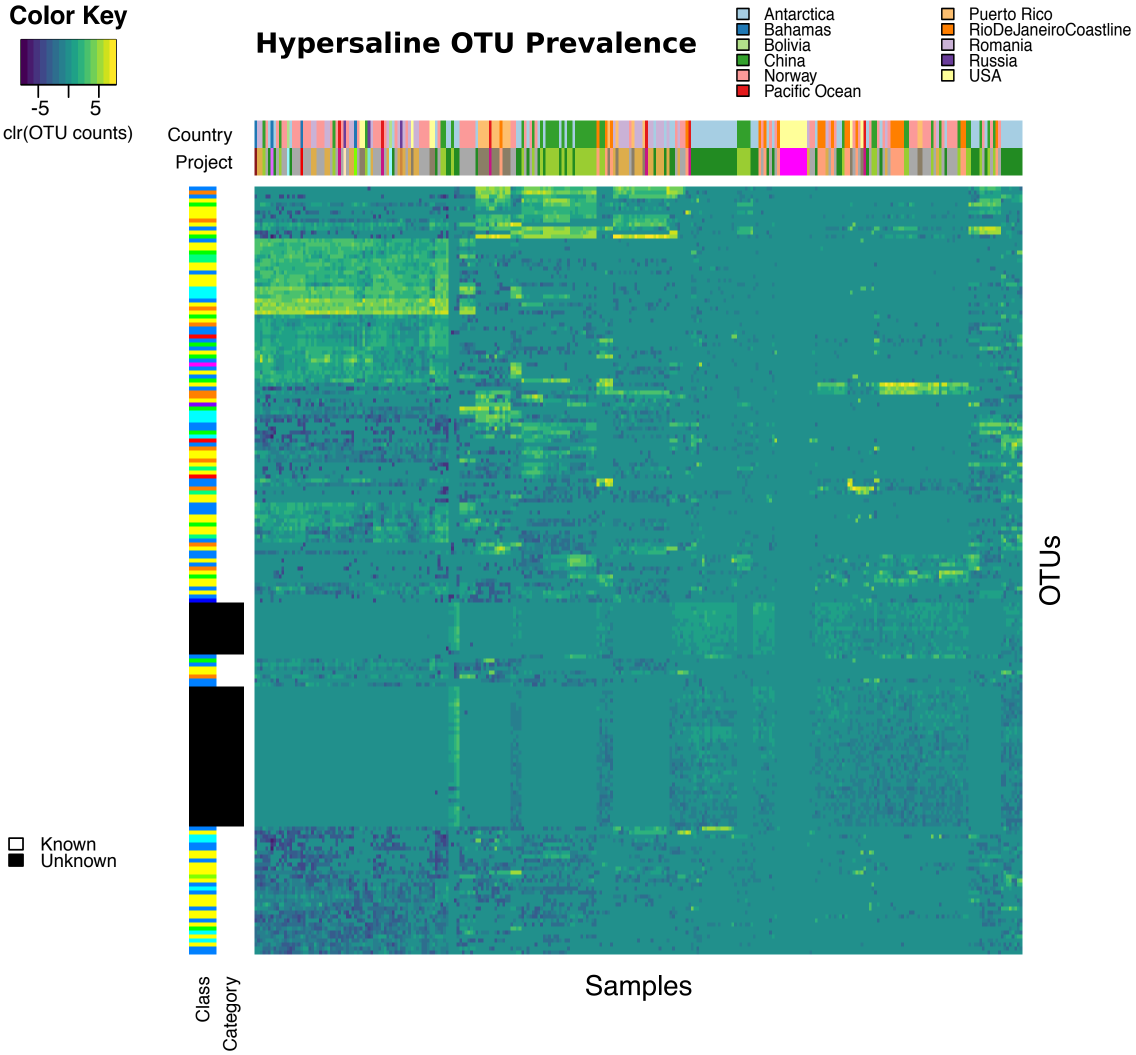

Supplementary Fig. S2. Taxa prevalence heatmap for hypersaline network. Relatve abundance (i.e., OTU counts) after centered-log-ratio (clr-)normalization, across all samples, is visualized using a blue-yellow color-scale mapping, with blue signifying low prevalence and yellow signifying high prevalence. By row, color bars depict OTU class and category (Unknown, black; Known,white). By column, color bars indicate sample contry and project source.

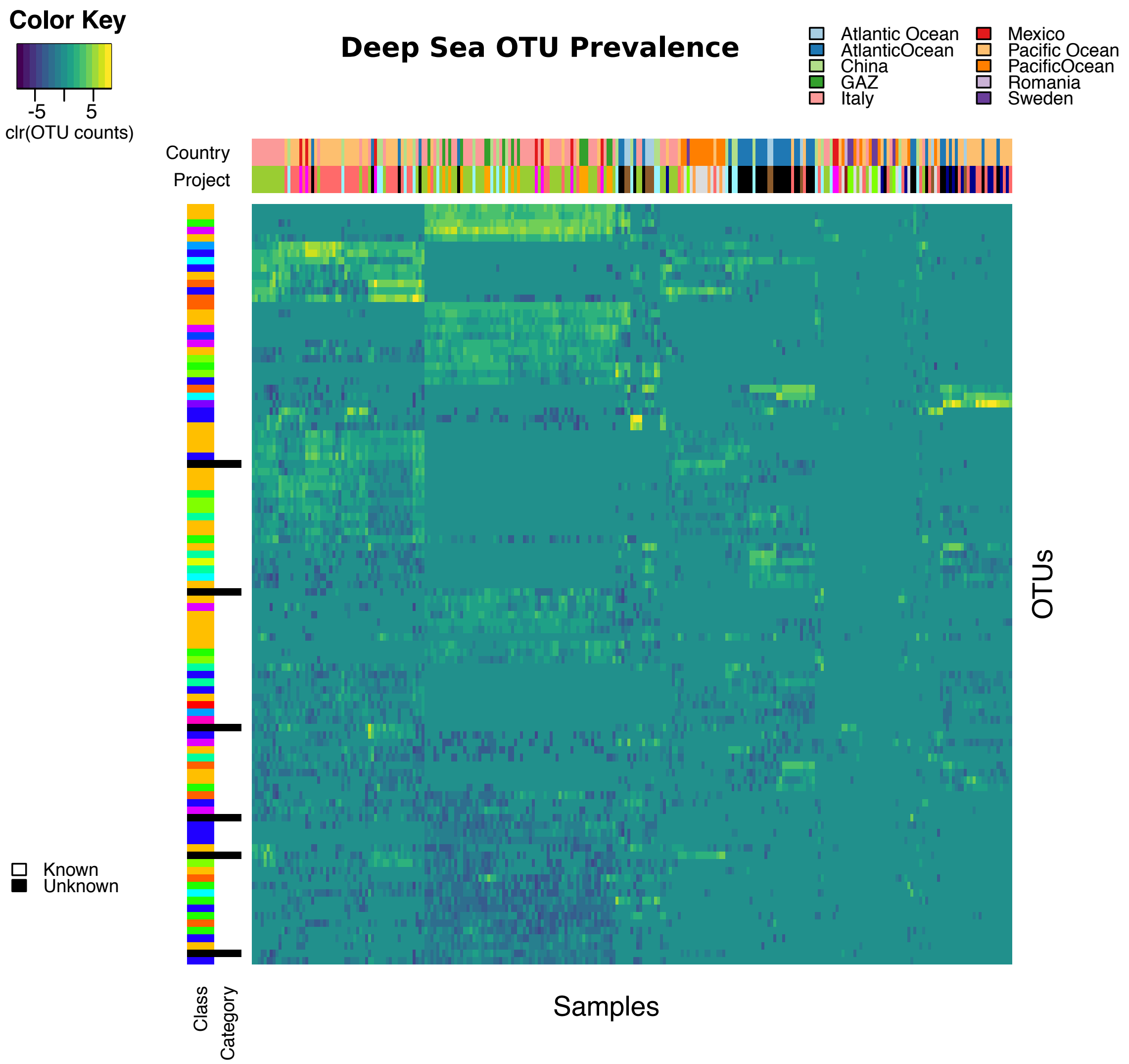

Supplementary Fig. S3. Taxa prevalence heatmap for deep sea network. Relatve abundance (i.e., OTU counts) after centered-log-ratio (clr-)normalization, across all samples, is visualized using a blue-yellow color-scale mapping, with blue signifying low prevalence and yellow signifying high prevalence. By row, color bars depict OTU class and category (Unknown, black; Known,white). By column, color bars indicate sample contry and project source.

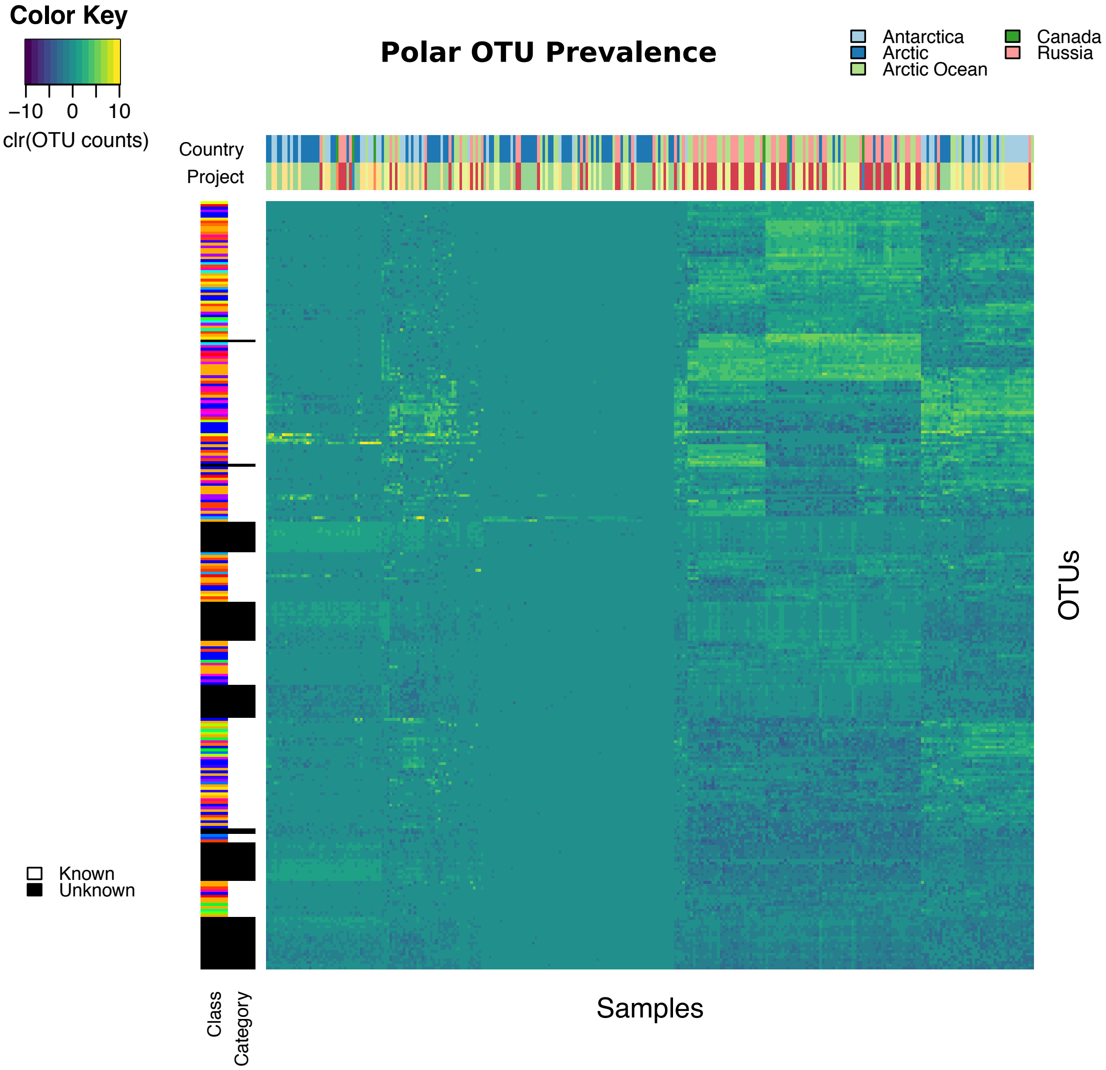

Supplementary Fig. S4. Taxa prevalence heatmap for polar network. Relatve abundance (i.e., OTU counts) after centered-log-ratio (clr)-normalization, across all samples, is visualized using a blue-yellow color-scale mapping, with blue signifying low prevalence and yellow signifying high prevalence. By row, color bars depict OTU class and category (Unknown, black; Known,white). By column, color bars indicate sample contry and project source.

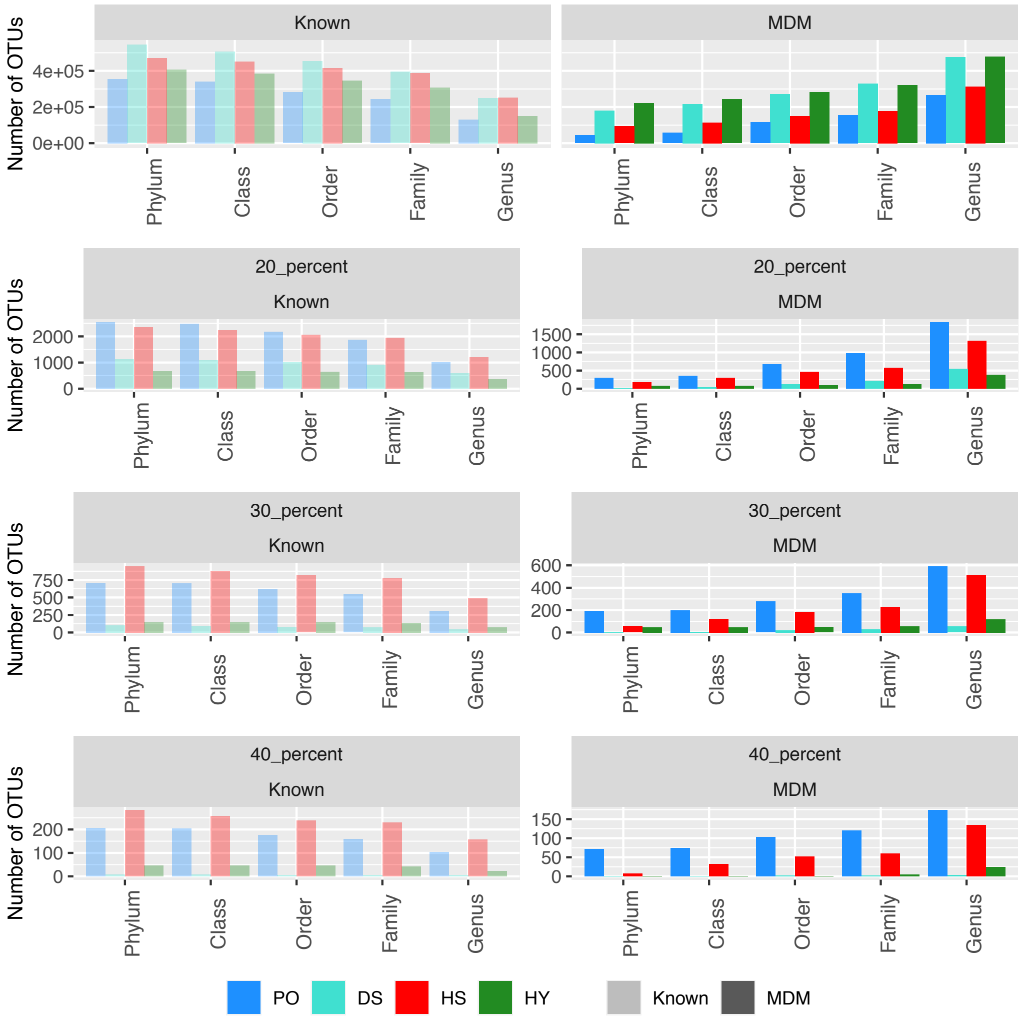

Supplementary Fig. S5. Number of Known to Unknown OTUs as a function of the prevalence threshold. Rows, from top to bottom, show the number of OTUs present in at least one, ≥ 20%, ≥30%, and ≥ 40% of all samples, across all taxonomic classification levels. Lighter shaded bars signify OTUs with known classification at each taxonomic level while darker shades represent microbial dark matter (MDM; unknown, ambiguous, or unassigned classification) at the same taxonomic level. PO: Polar, DS: Deep sea, HS: Hot springs, HY: Hypersaline.

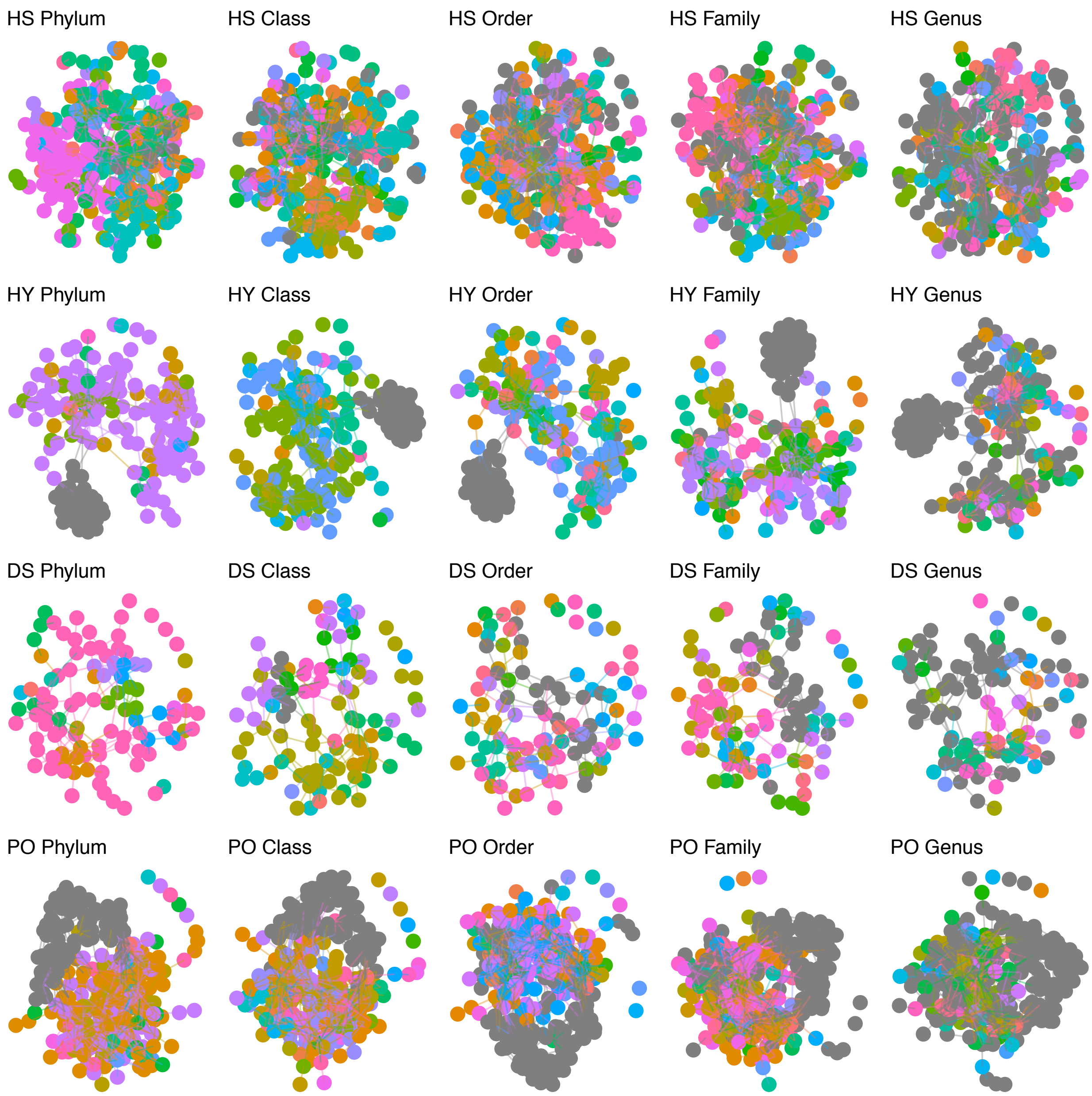

Supplementary Fig. S6. Extreme environmental networks across taxonomic levels. Each environment is represented in a different row, while taxonomic levels are arranged by column. Nodes in each network indicate different taxa at each taxonomic level. Nodes with no designated classification at the indicated taxonomic level are colored gray and represent the unknown taxa at the level. Within each environment, similar distribution of unknown OTUs are observed regardless the taxonomic level. PO: Polar, DS: Deep sea, HS: Hot springs, HY: Hypersaline.

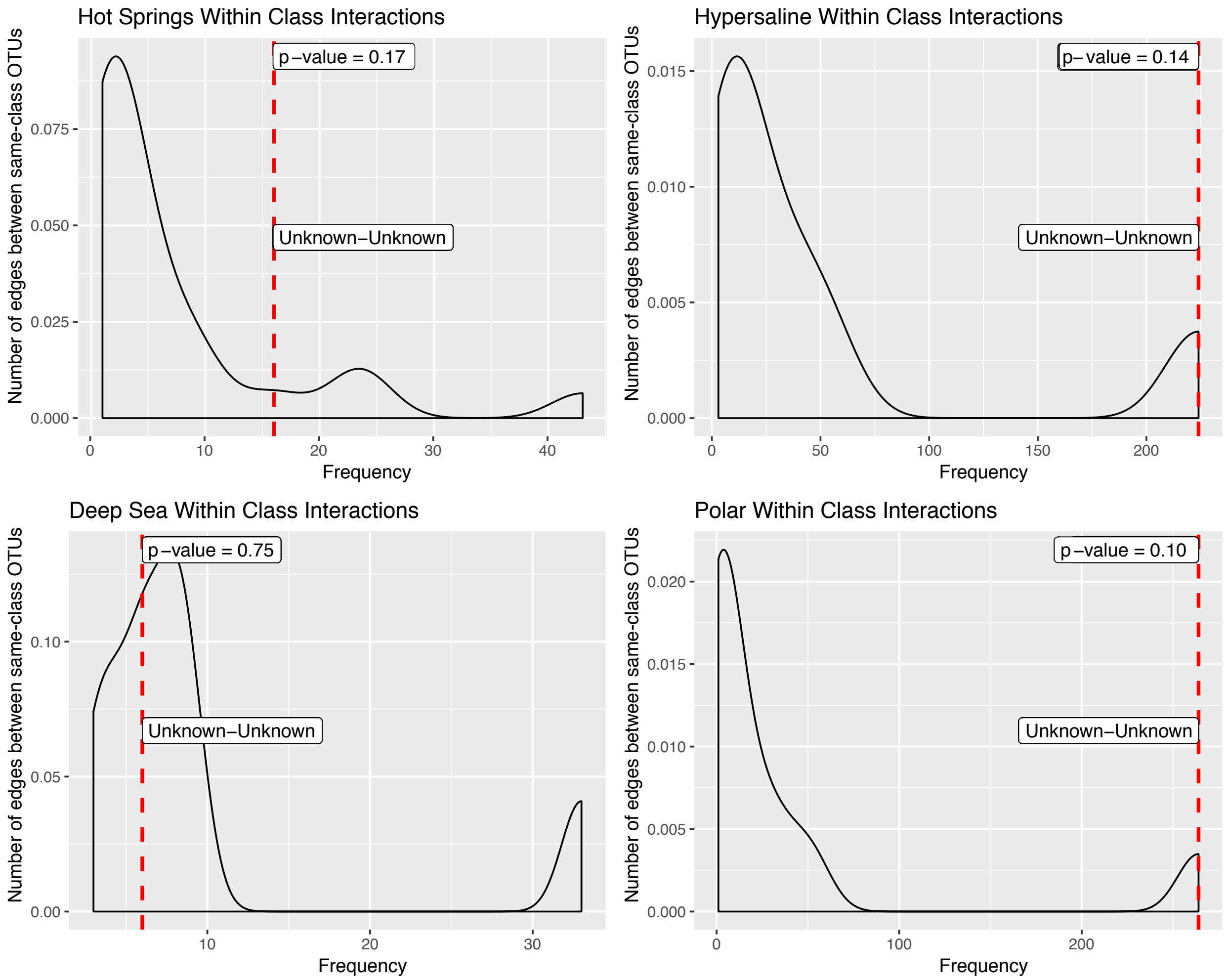

Supplementary Fig. S7. Density plots of within-class OTU interactions. The density plot represents the distribution of the number of edges joining two OTUs from the same class, across all classes detected in the environment. The dotted red line indicates the value for number of edges linking two unknown OTUs. The empirical p-value of this number with respect to the density distribution is indicated.

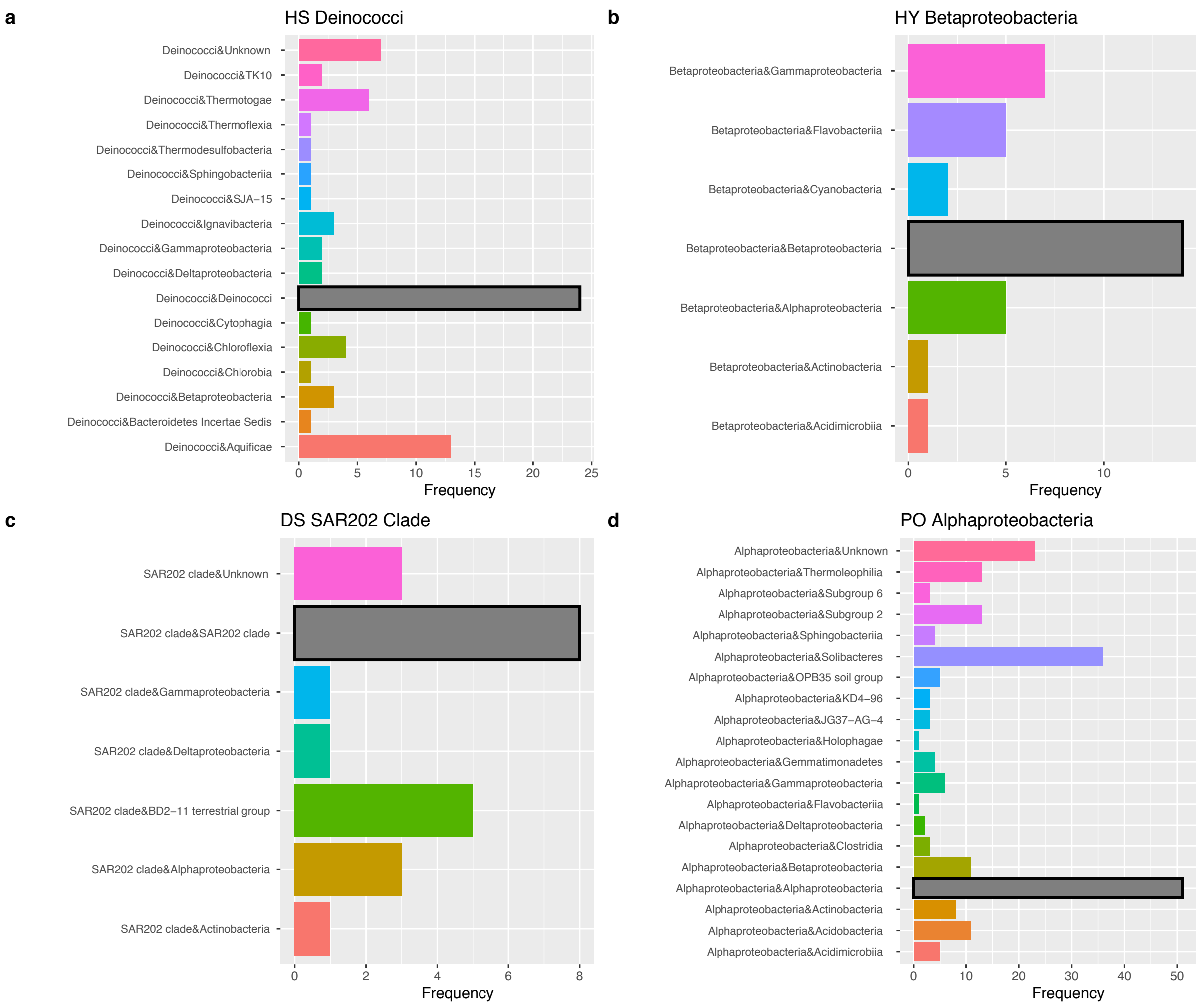

Supplementary Fig. S8. Bar graphs of the co-occurrence relationships (i.e., edges) of known OTUs with other taxa at the class-level within each environmental network. **a** Co-occurrence relationships of *Deinococci* with other taxa present in Hot Springs (HS)class network. **b** Co-occurrence relationships of *Betaproteobacteria with* other taxa present in Hypersaline (HY) class network. **c** Co-occurrence relationships of SAR202 Clade with other taxa present in Deep Sea (DS) class network. **d** Co-occurrence relationships of *Alphaproteobacteria* with other taxa present in Polar (PO) class network. Y-axis labels and colors signify the different classes with which unknowns were found to co-occur. Within-class relationships are represented in gray.

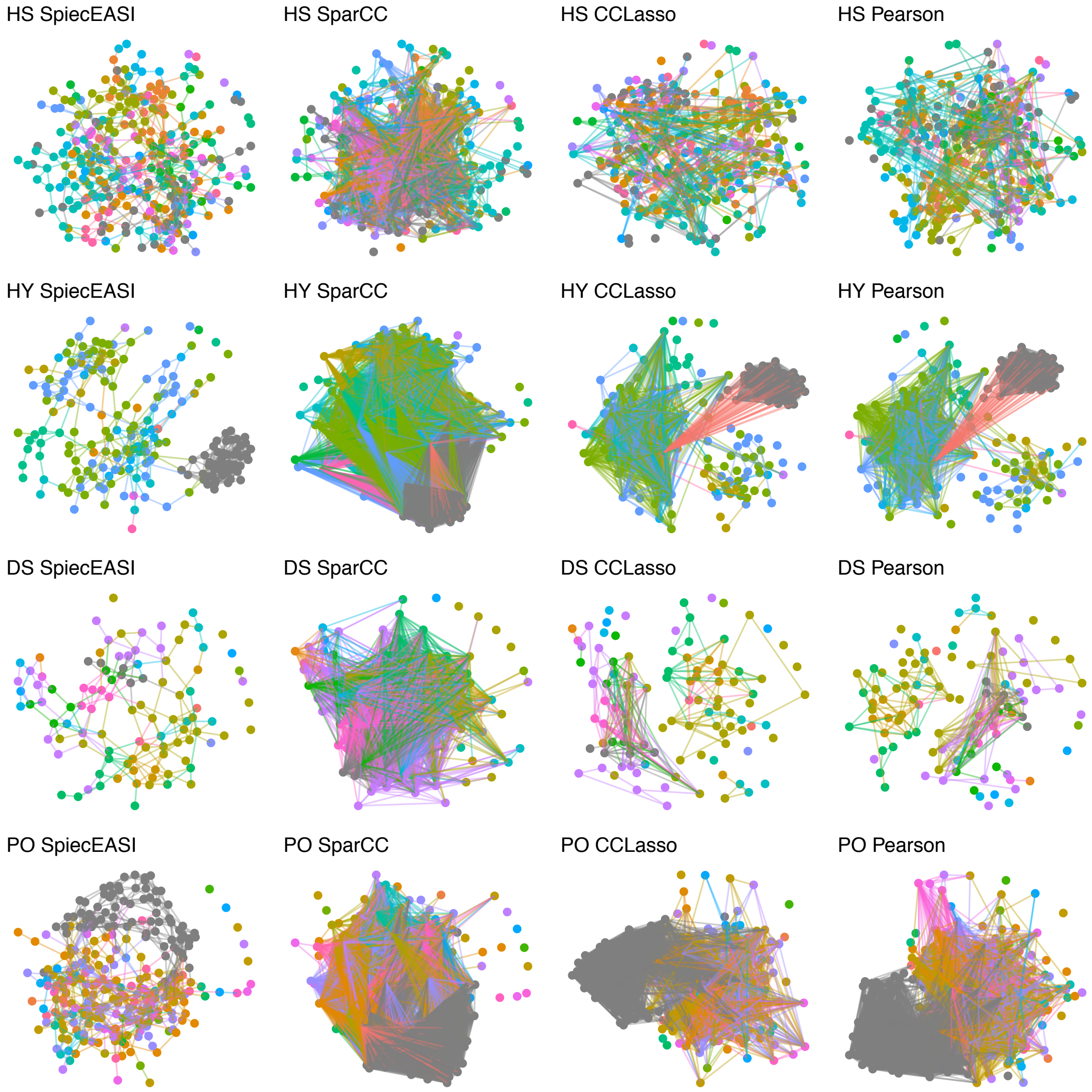

Supplementary Fig. S9. Environmental networks at class-level constructed with different correlation and regression methods. Each environment is represented in a different row, while network construction algorithms are arranged by column. Nodes in each network indicate different taxa at each taxonomic level. Nodes with unknown classification are colored gray. See Methods for parameter choices. Within each environment, similar distribution of unknown OTUs are observed regardless the algorithm used. PO: Polar, DS: Deep sea, HS: Hot springs, HY: Hypersaline.

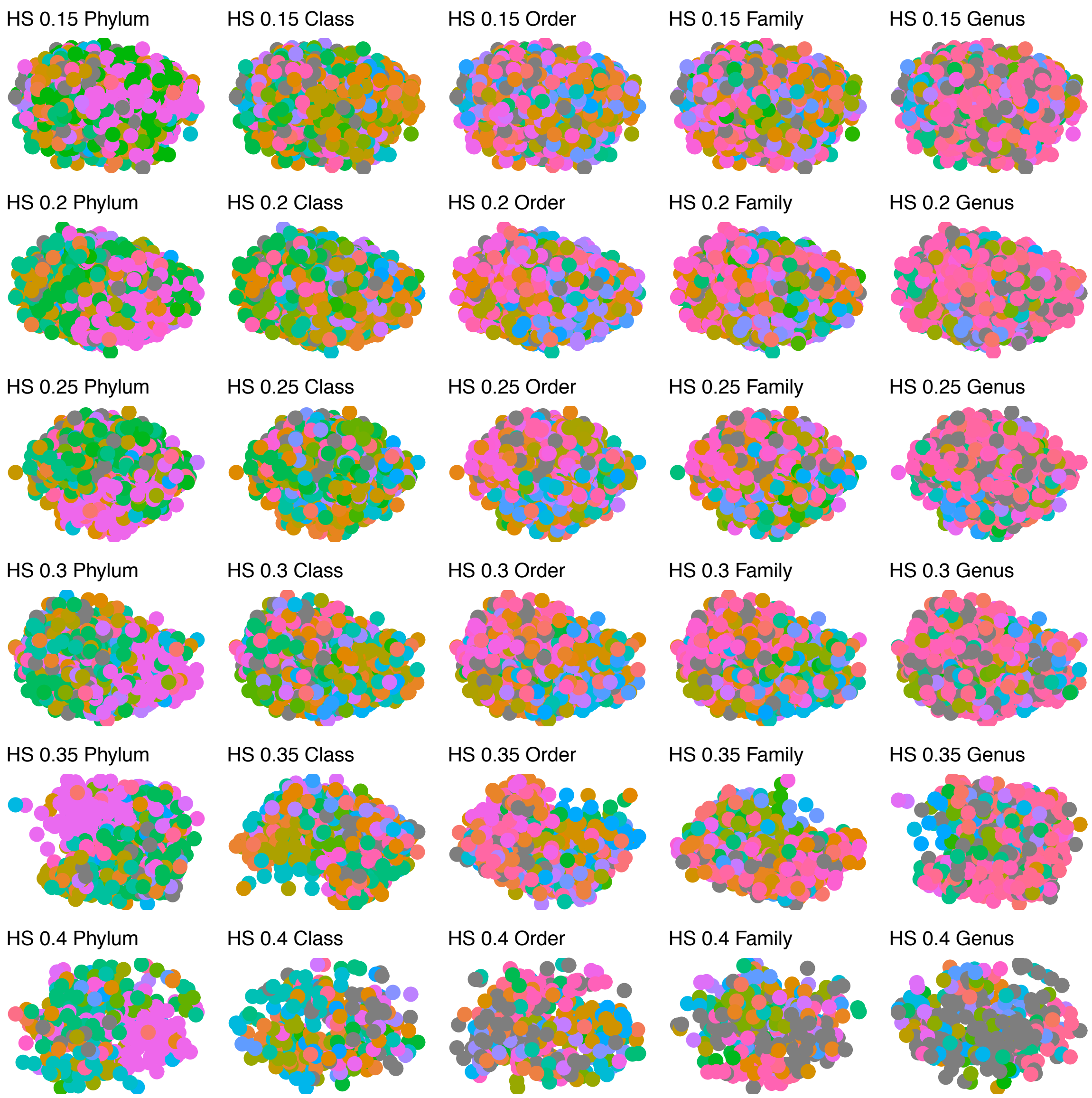

Supplementary Fig. S10. Networks of targeted hot springs (HS) environments at different prevalence thresholds and taxonomic levels. Prevalence thresholds are arranged by row, while taxonomic levels are arranged by column. Last row indicates highest prevalence threshold for which a network could still be produced. Nodes in each network indicate different taxa at each taxonomic level. Nodes with no designated classification at the indicated taxonomic level are colored gray and represent the unknown taxa at the level. Similar network topology is observed across threshold levels.

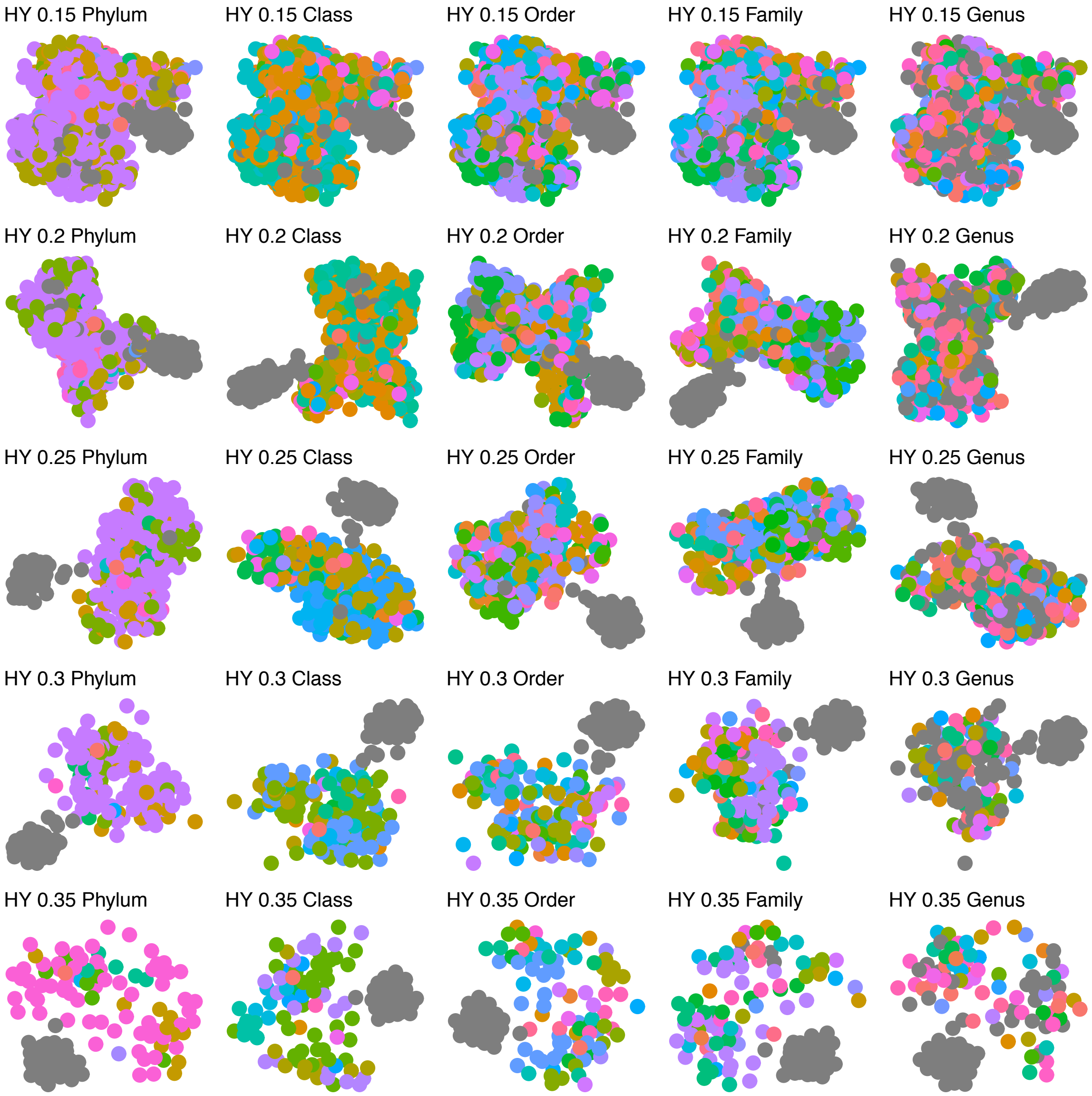

Supplementary Fig. S11. Networks of targeted hypersaline (HY) environments at different prevalence thresholds and taxonomic levels. Prevalence thresholds are arranged by row, while taxonomic levels are arranged by column. Last row indicates highest prevalence threshold for which a network could still be produced. Nodes in each network indicate different taxa at each taxonomic level. Nodes with no designated classification at the indicated taxonomic level are colored gray and represent the unknown taxa at the level. Similar network topology is observed across threshold levels.

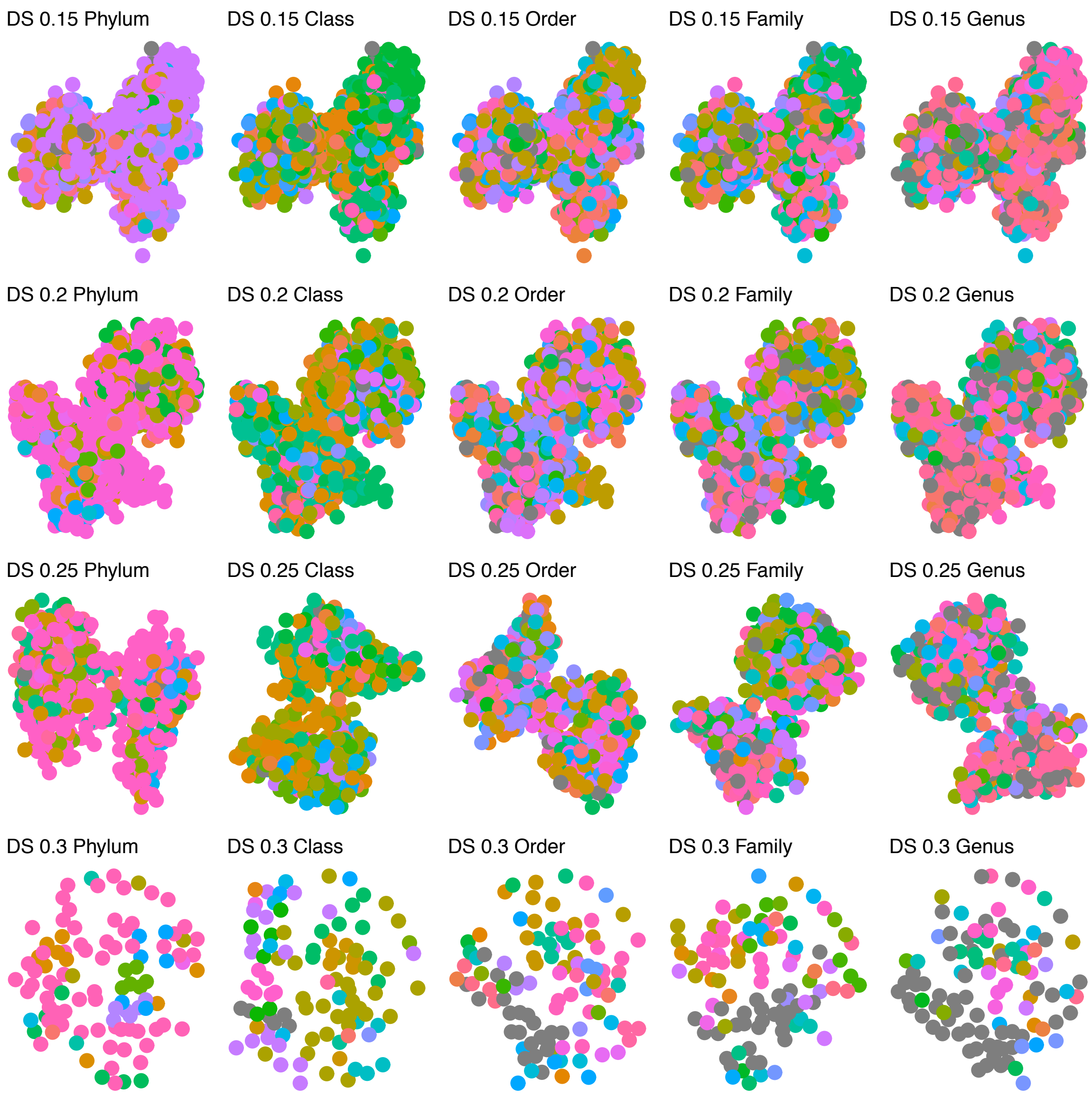

Supplementary Fig. S12. Networks of targeted deep sea (DS) environments at different prevalence thresholds and taxonomic levels. Prevalence thresholds are arranged by row, while taxonomic levels are arranged by column. Last row indicates highest prevalence threshold for which a network could still be produced. Nodes in each network indicate different taxa at each taxonomic level. Nodes with no designated classification at the indicated taxonomic level are colored gray and represent the unknown taxa at the level. Similar network topology is observed across threshold levels

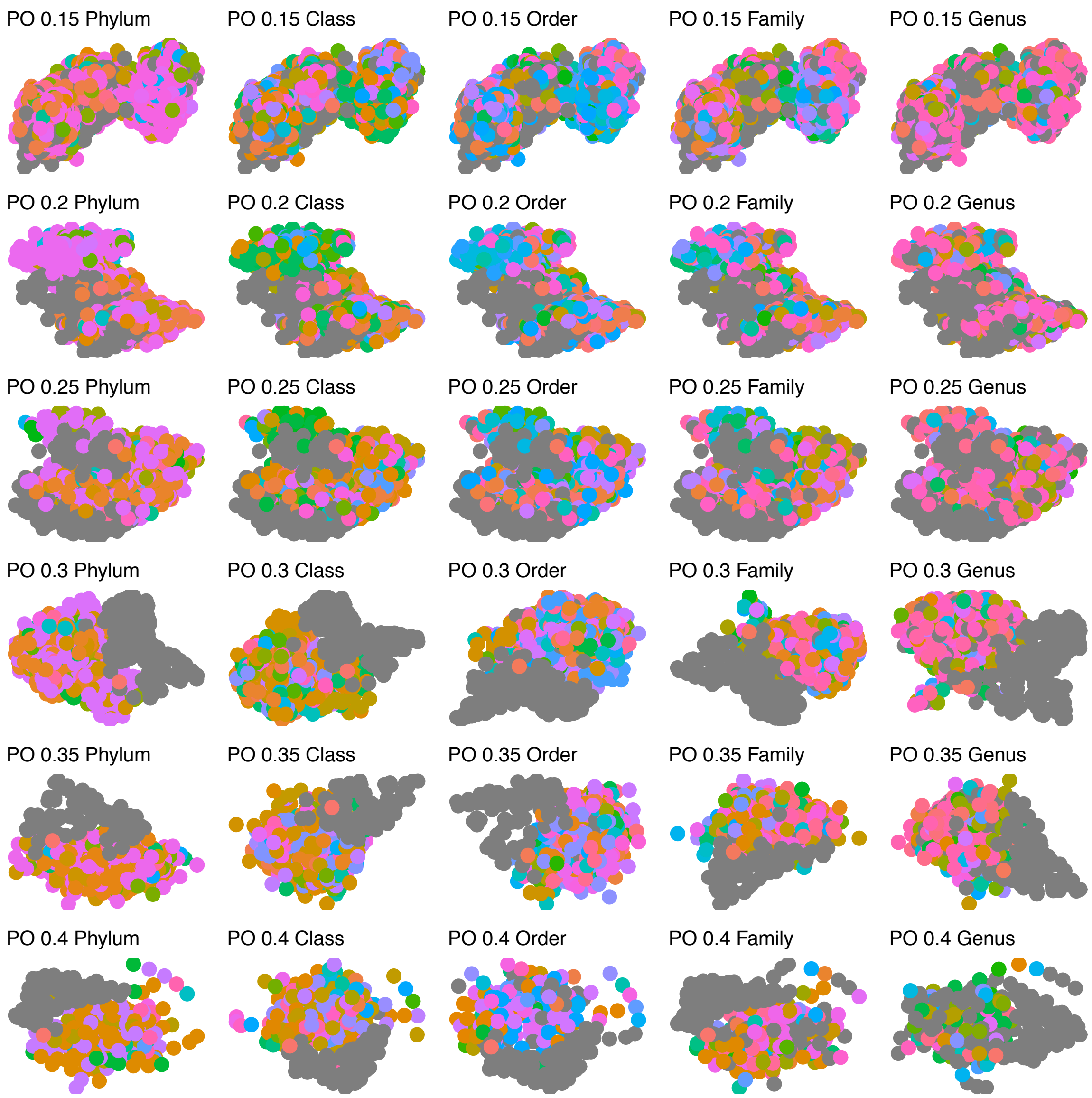

Supplementary Fig. S13. Networks of targeted polar (PO) environments at different prevalence thresholds and taxonomic levels. Prevalence thresholds are arranged by row, while taxonomic levels are arranged by column. Last row indicates highest prevalence threshold for which a network could still be produced. Nodes in each network indicate different taxa at each taxonomic level. Nodes with no designated classification at the indicated taxonomic level are colored gray and represent the unknown taxa at the level. Similar network topology is observed across threshold levels.

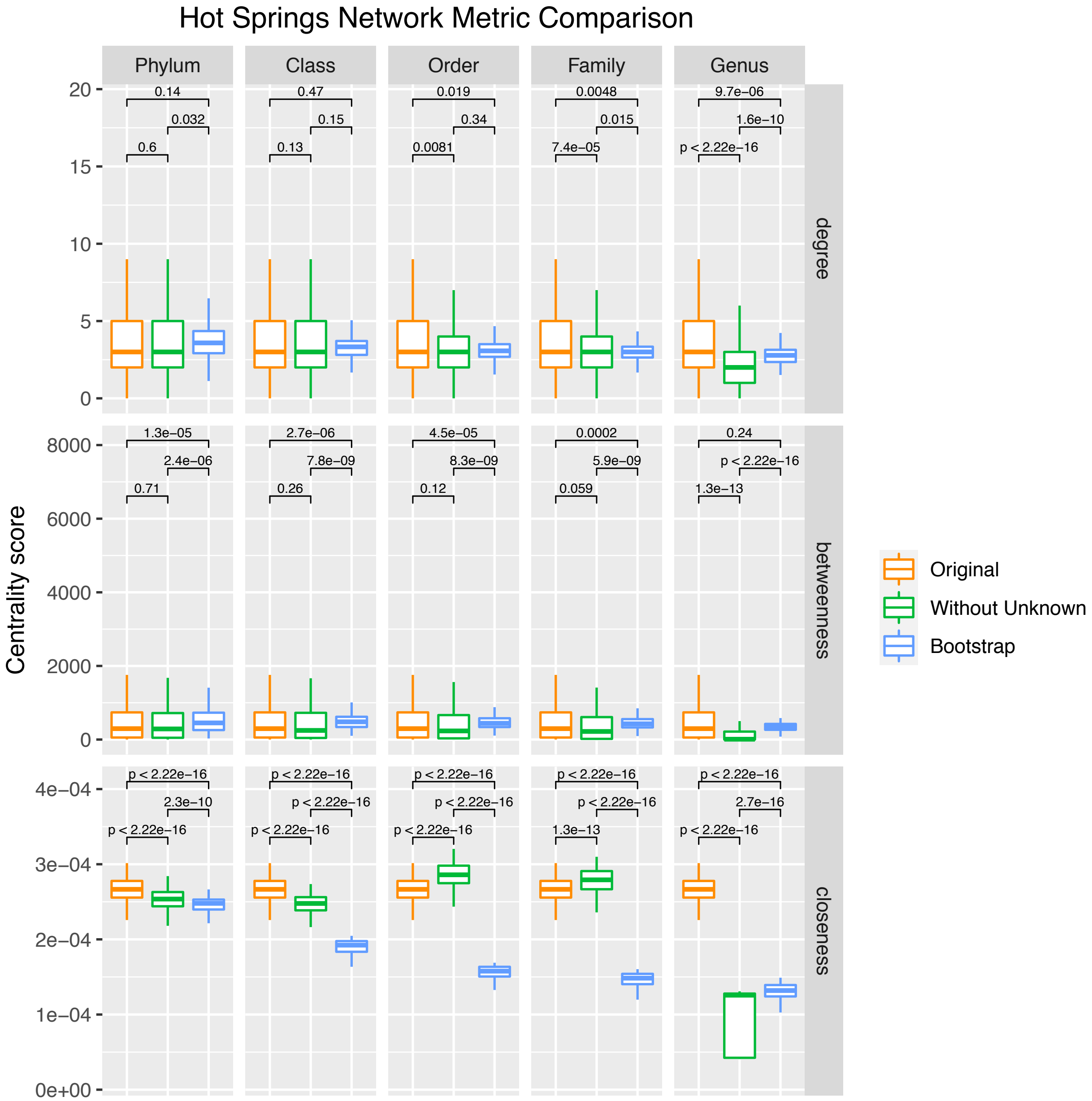

Supplementary Fig. S14. Impact of unknown taxa on hot springs network measures at different taxonomic levels. From top to bottom, boxplots depicting degree, betweenness, and closeness centrality measures of nodes present within the Original (orange), Without Unknown (light green), and Bootstrap networks (light blue) at different taxonomic levels. Wilcoxon pairwise comparisons were used to assess significance between the three network types (Original-Without Unknown, Without Unknown-Bootstrap, Original-Bootstrap) for each taxonomic level. For each pairwise comparison, p-values after Holm adjustment are shown.

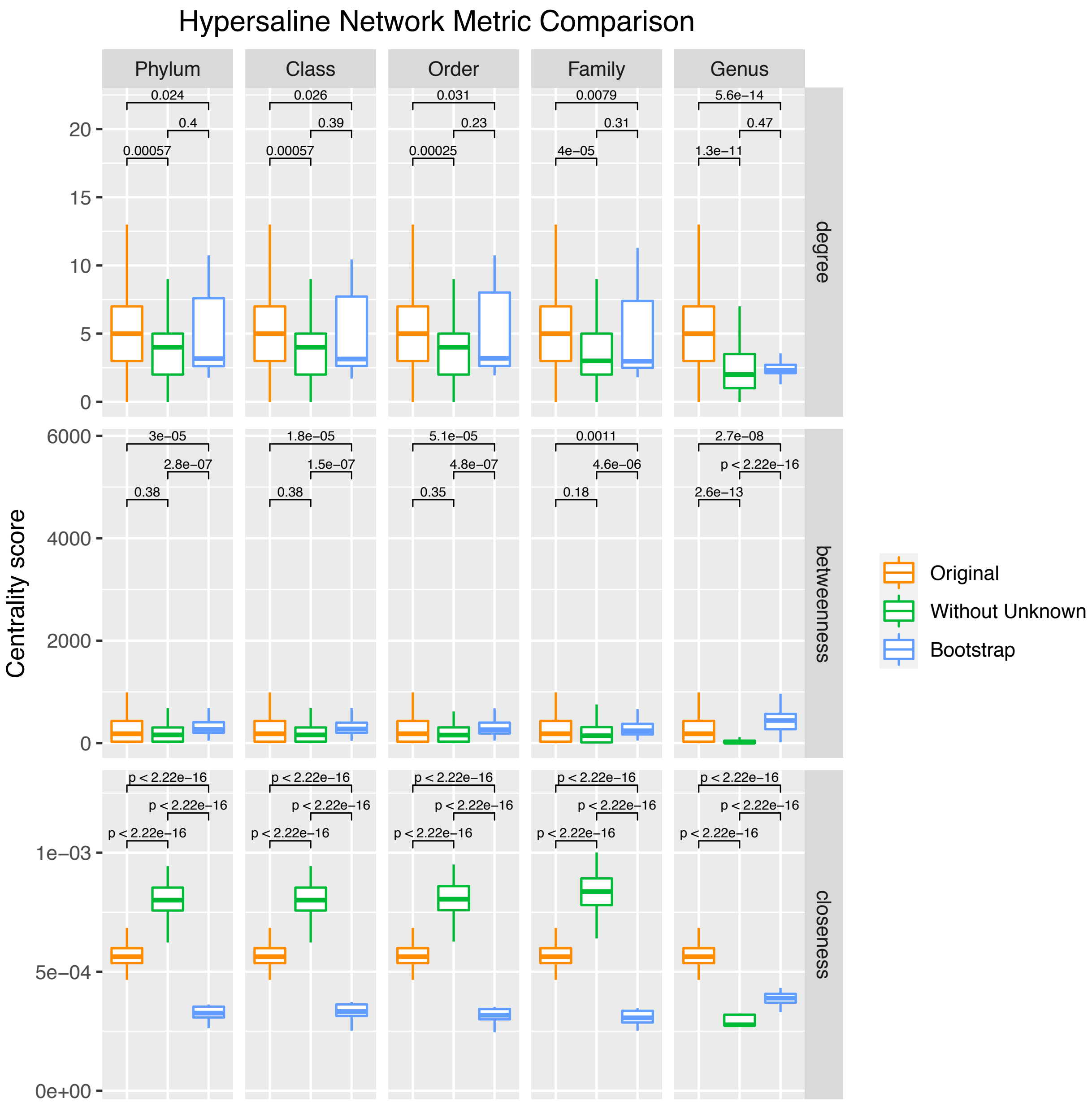

Supplementary Fig. S15. Impact of unknown taxa on hypersaline network measures at different taxonomic levels. From top to bottom, boxplots depicting degree, betweenness, and closeness centrality measures of nodes present within the Original (orange), Without Unknown (light green), and Bootstrap networks (light blue) at different taxonomic levels. Wilcoxon pairwise comparisons were used to assess significance between the three network types (Original-Without Unknown, Without Unknown-Bootstrap, Original-Bootstrap) for each taxonomic level. For each pairwise comparison, p-values after Holm adjustment are shown.

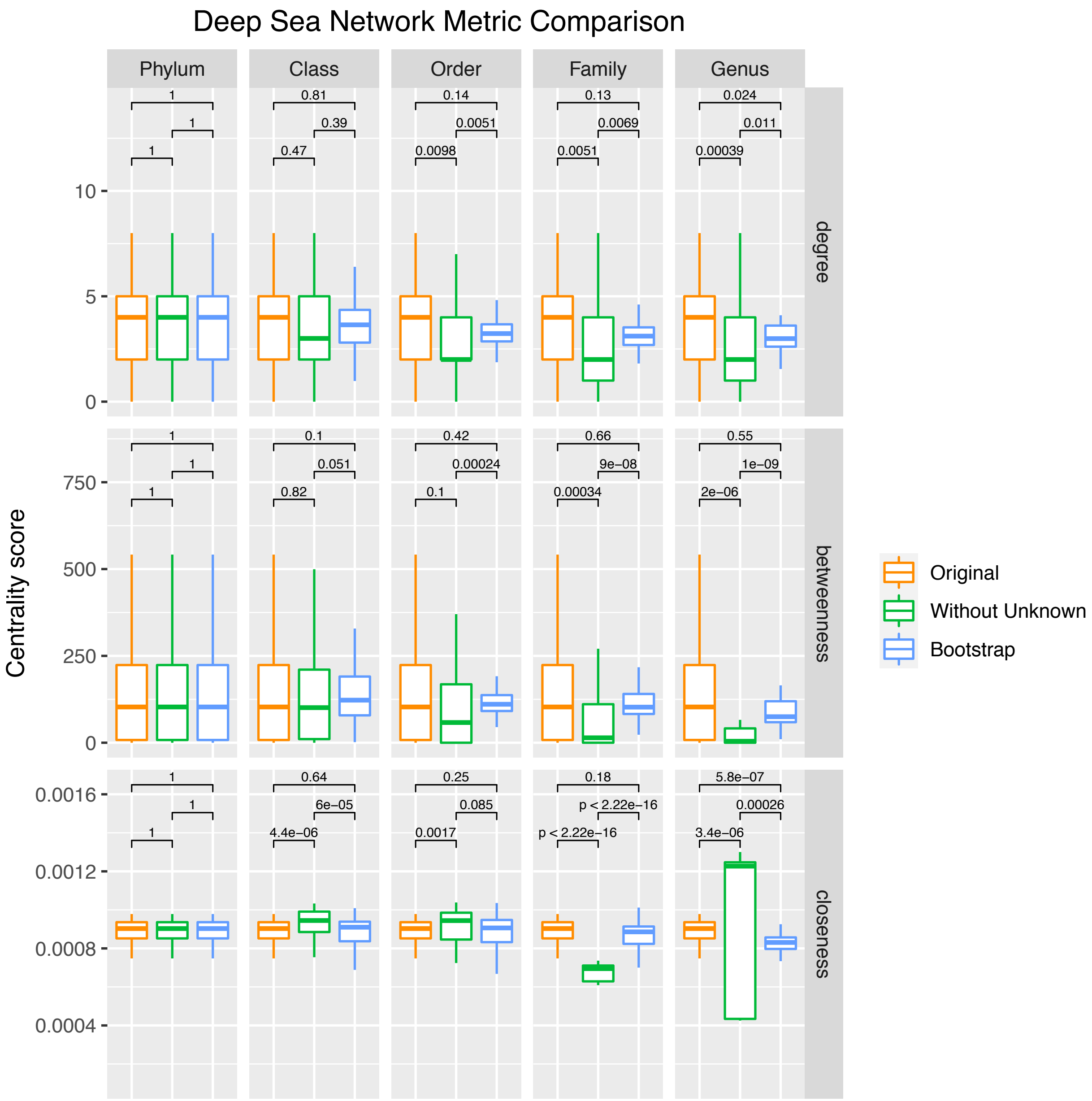

Supplementary Fig. S16. Impact of unknown taxa on deep sea network measures at different taxonomic levels. From top to bottom, boxplots depicting degree, betweenness, and closeness centrality measures of nodes present within the Original (orange), Without Unknown (light green), and Bootstrap networks (light blue) at different taxonomic levels. Wilcoxon pairwise comparisons were used to assess significance between the three network types (Original-Without Unknown, Without Unknown-Bootstrap, Original-Bootstrap) for each taxonomic level. For each pairwise comparison, p-values after Holm adjustment are shown.

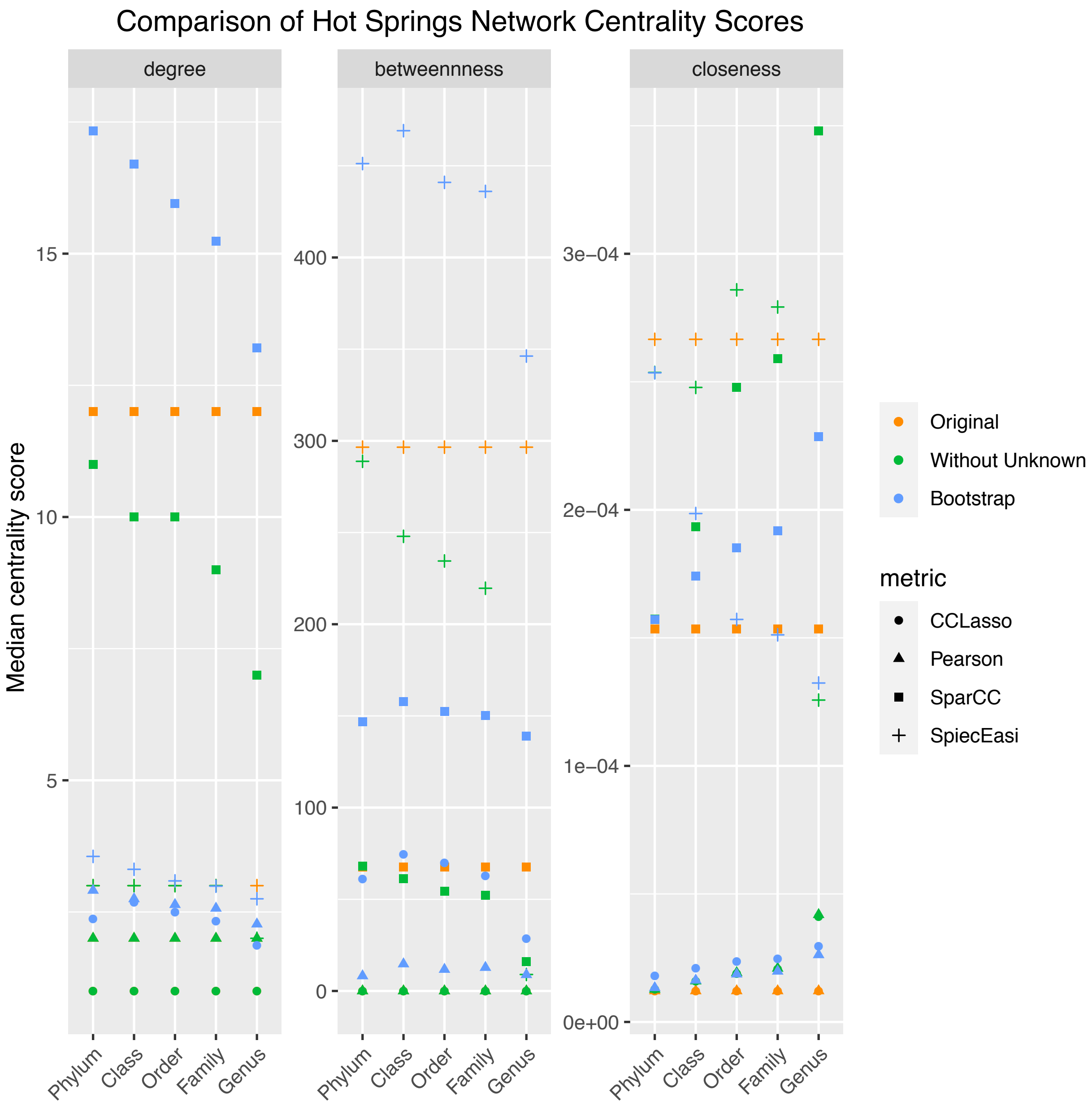

Supplementary Fig. S17. Comparison of hot springs network centrality scores across correlation metric tools. Median value of degree, betweenness, and closeness centrality scores for different hot springs network types and network construction algorithms. The direction of centrality score changes among network types is consistent across algorithms. Shapes delineate correlation metric used (CCLasso, circle; Pearson, triangle; SparCC, square; SpiecEasi, plus sign). Colors signify network type (Original, orange; Without Unknown, green, Bootstrap, blue) across taxonomic levels.

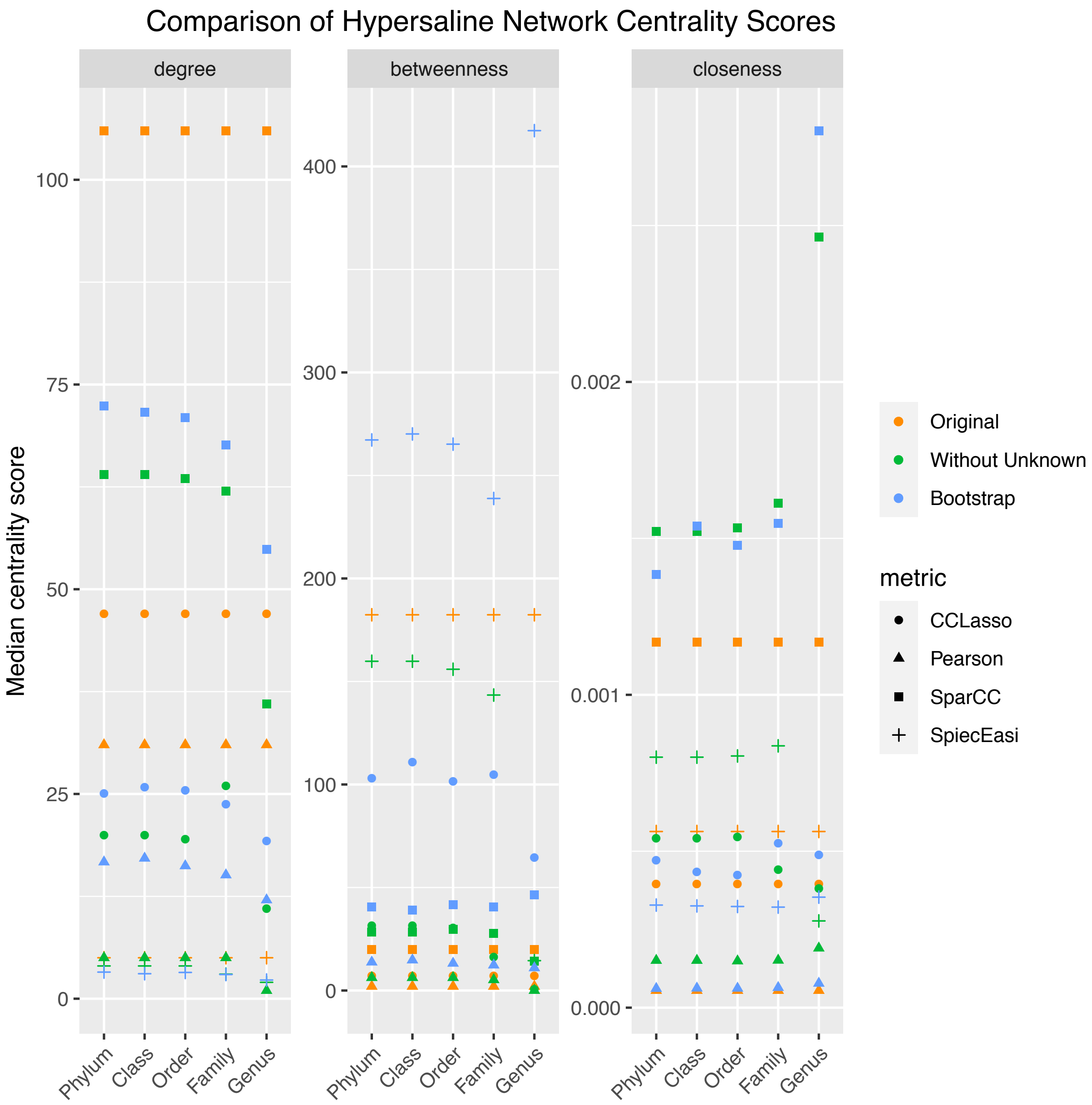

Supplementary Fig. S18. Comparison of hypersaline network centrality scores across correlation metric tools. Median value of degree, betweenness, and closeness centrality scores for different hypersaline network types and network construction algorithms. The direction of centrality score changes among network types is consistent across algorithms. Shapes delineate correlation metric used (CCLasso, circle; Pearson, triangle; SparCC, square; SpiecEasi, plus sign). Colors signify network type (Original, orange; Without Unknown, green, Bootstrap, blue) across taxonomic levels.

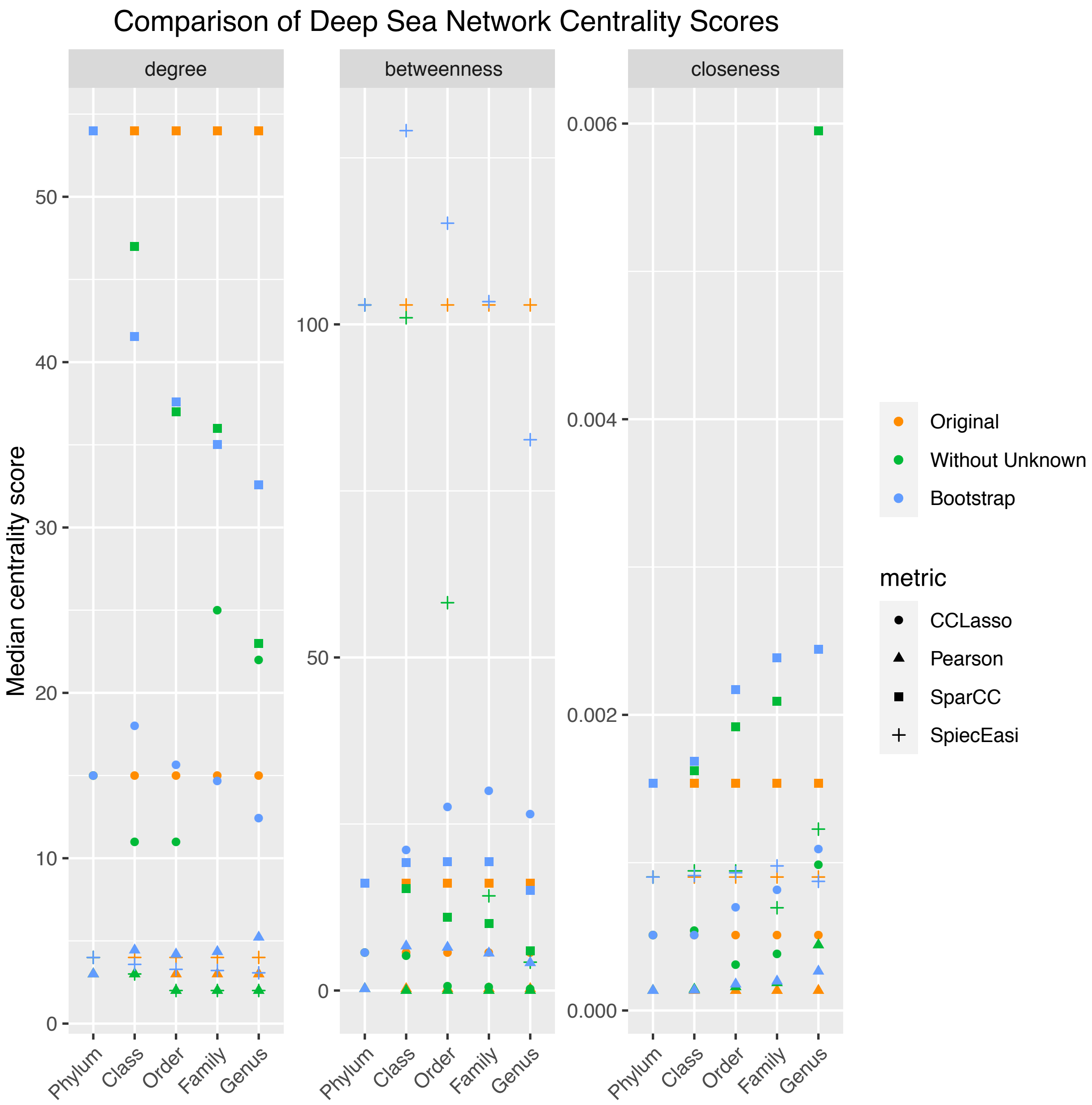

Supplementary Fig. S19. Comparison of deep sea network centrality scores across correlation metric tools. Median value of degree, betweenness, and closeness centrality scores for different deep sea network types and network construction algorithms. The direction of centrality score changes among network types is consistent across algorithms. Shapes delineate correlation metric used (CCLasso, circle; Pearson, triangle; SparCC, square; SpiecEasi, plus sign). Colors signify network type (Original, orange; Without Unknown, green, Bootstrap, blue) across taxonomic levels.

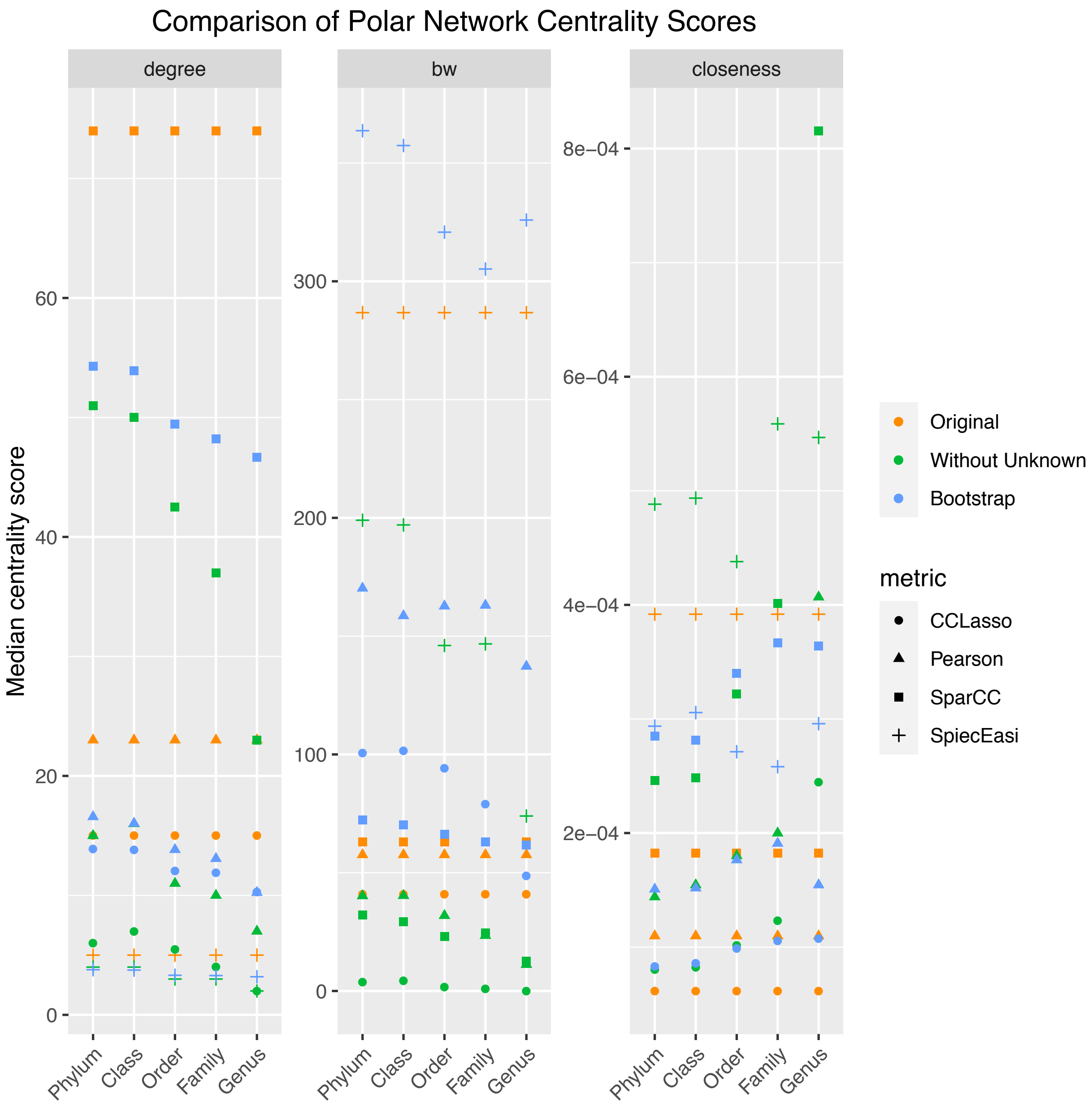

Supplementary Fig. S20. Comparison of polar network centrality scores across correlation metric tools. Median value of degree, betweenness, and closeness centrality scores for different polar network types and network construction algorithms. The direction of centrality score changes among network types is consistent across algorithms. Shapes delineate correlation metric used (CCLasso, circle; Pearson, triangle; SparCC, square; SpiecEasi, plus sign). Colors signify network type (Original, orange; Without Unknown, green, Bootstrap, blue) across taxonomic levels.

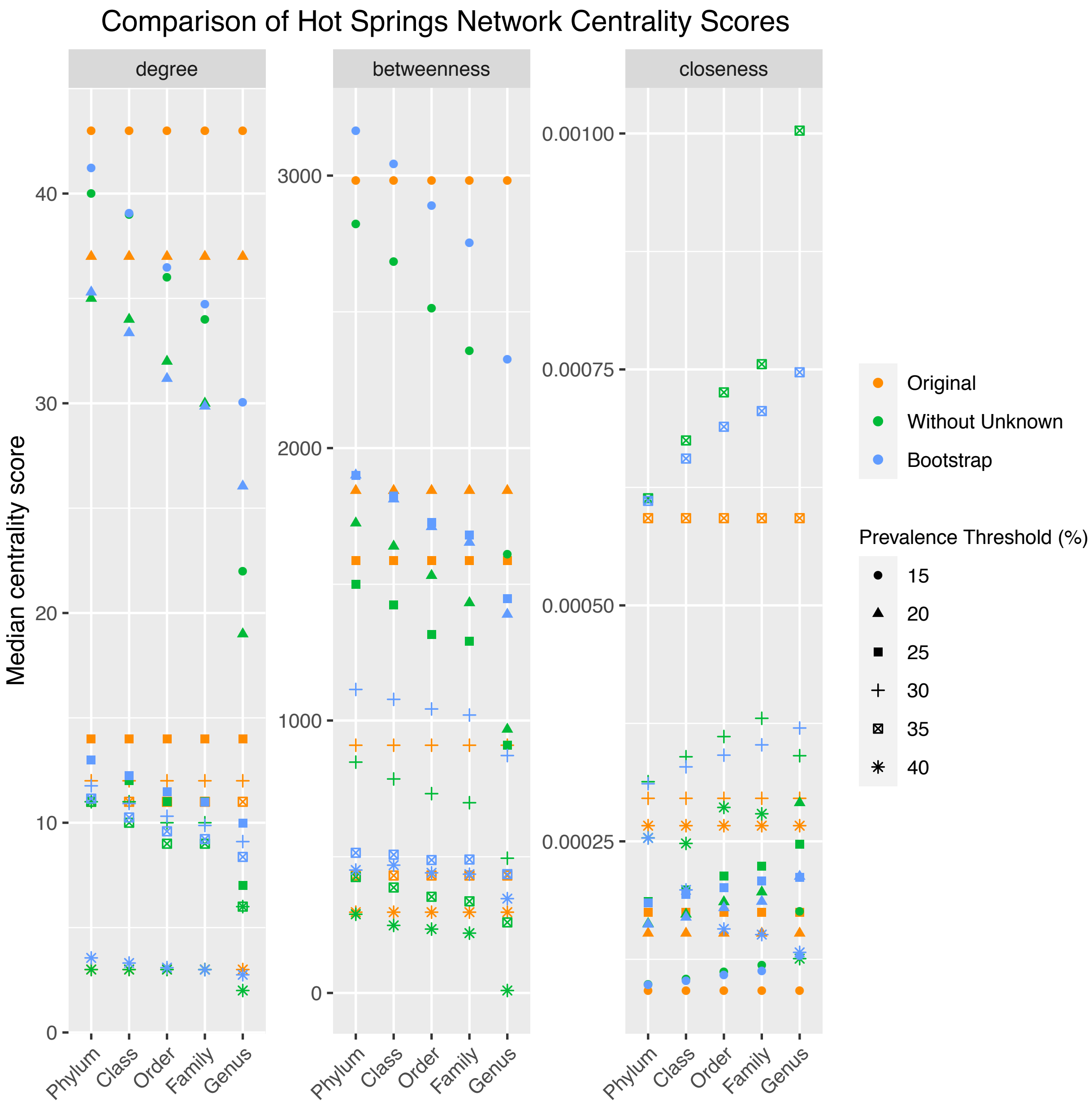

Supplementary Fig. S21. Comparison of hot springs network centrality scores across prevalence thresholds. Median value of degree, betweenness, and closeness centrality scores for different hot springs network types and prevalence thresholds. The direction of centrality score changes among network types is consistent prevalence thresholds. Different shapes signify different percent sample threshold criteria and colors signify network type (Original, orange; Without Unknown, green; Bootstrap, blue) for each taxonomic level.

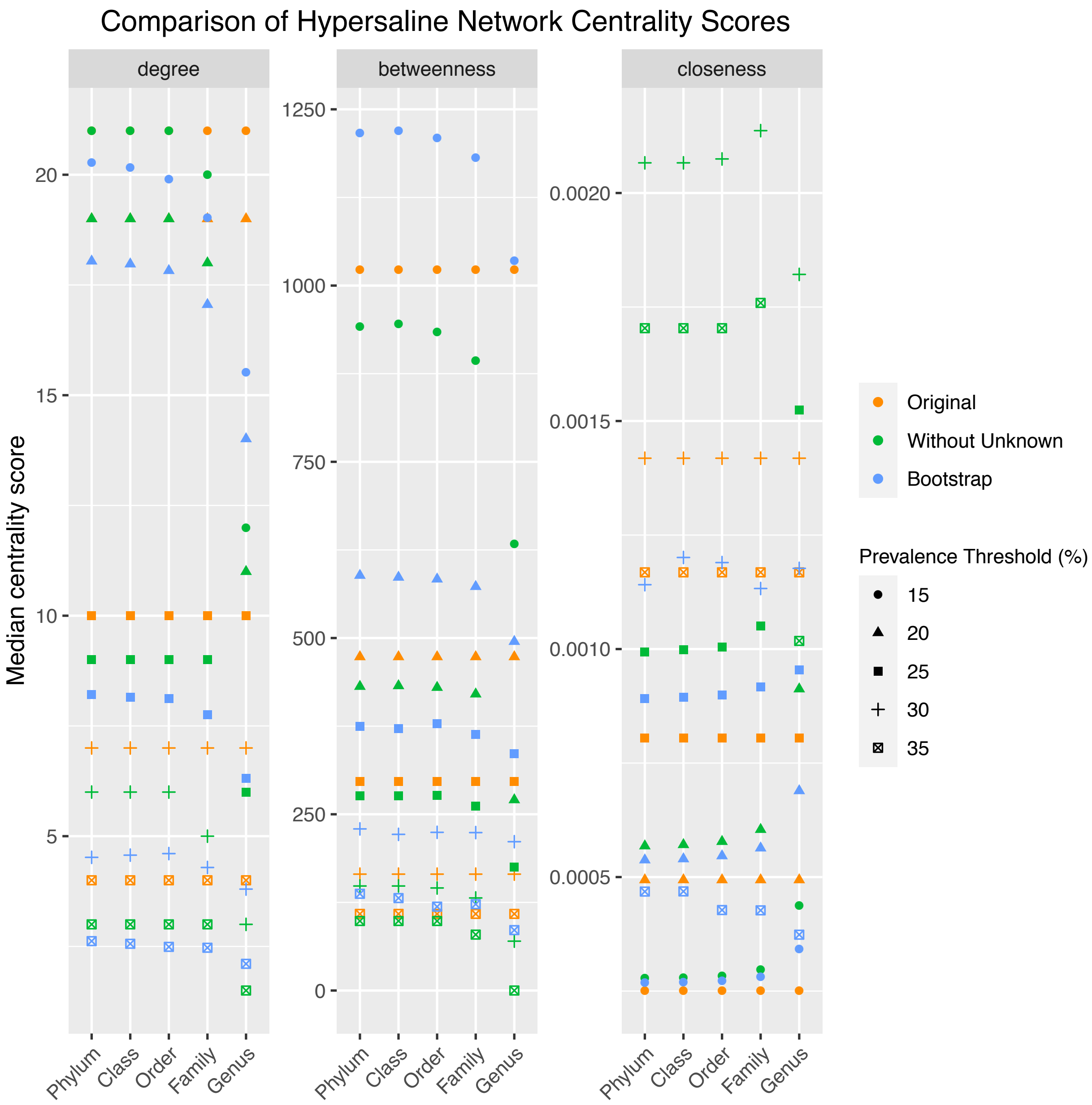

Supplementary Fig. S22. Comparison of hypersaline network centrality scores across prevalence thresholds. Median value of degree, betweenness, and closeness centrality scores for different hypersaline network types and prevalence thresholds. The direction of centrality score changes among network types is consistent prevalence thresholds.   Different shapes signify different percent sample threshold criteria and colors signify network type (Original, orange; Without Unknown, green; Bootstrap, blue) for each taxonomic level.

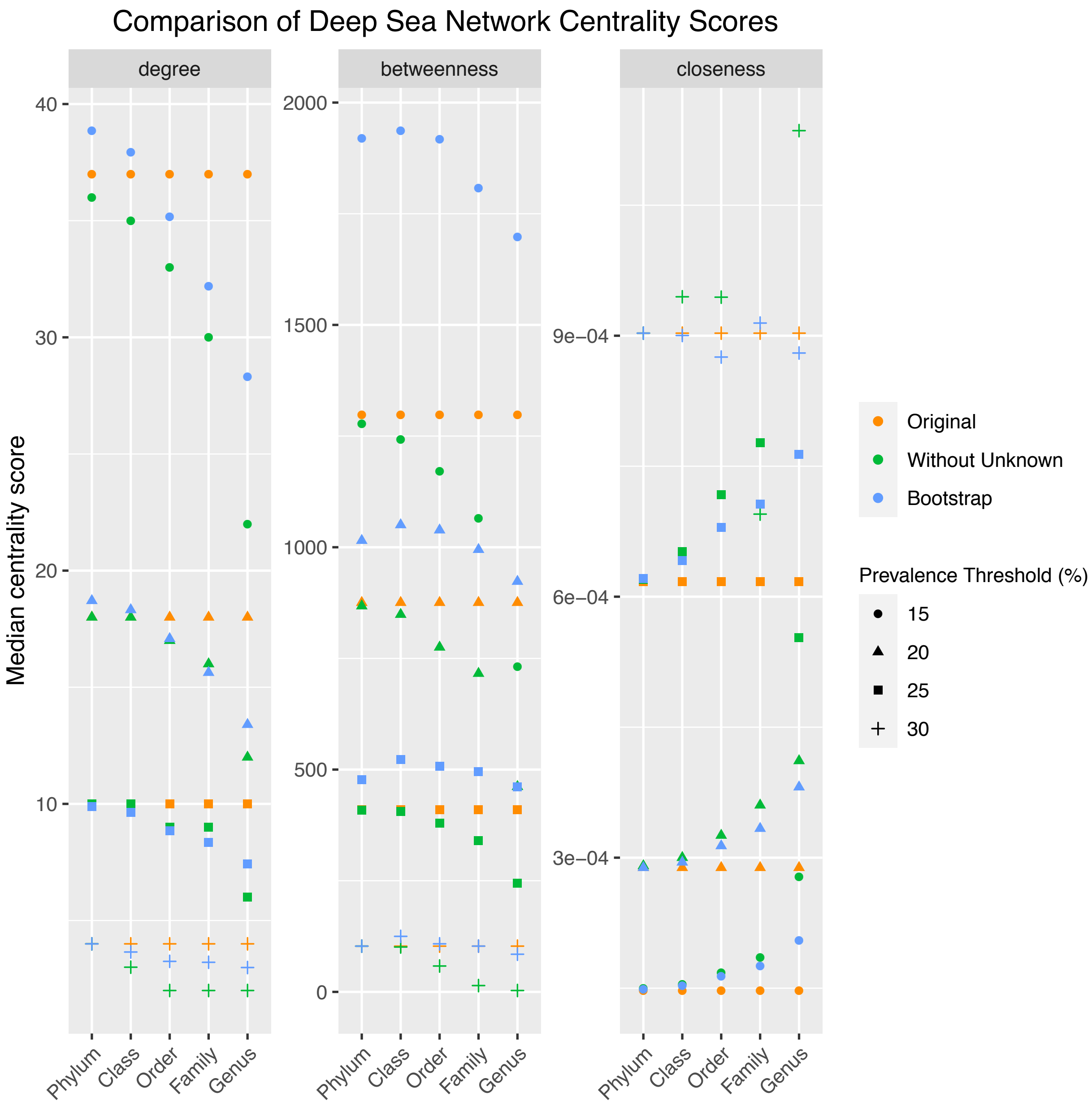

Supplementary Fig. S23. Comparison of deep sea network centrality scores across prevalence thresholds. Median value of degree, betweenness, and closeness centrality scores for different deep sea network types and prevalence thresholds. The direction of centrality score changes among network types is consistent prevalence thresholds. Different shapes signify different percent sample threshold criteria and colors signify network type (Original, orange; Without Unknown, green; Bootstrap, blue) for each taxonomic level.

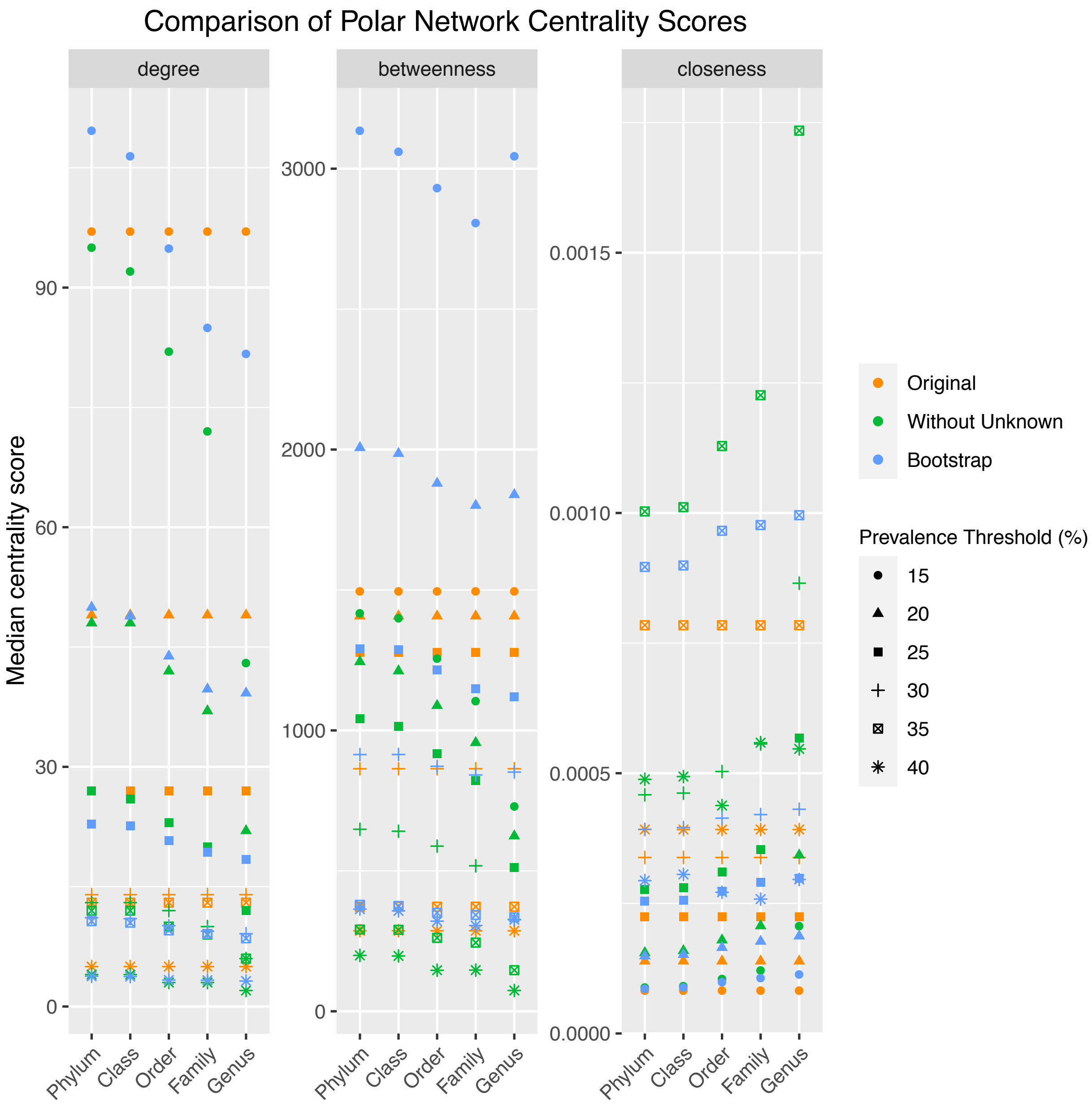

Supplementary Fig. S24. Comparison of polar network centrality scores across prevalence thresholds. Median value of degree, betweenness, and closeness centrality scores for different polar network types and prevalence thresholds. The direction of centrality score changes among network types is consistent prevalence thresholds. Different shapes signify different percent sample threshold criteria and colors signify network type (Original, orange; Without Unknown, green; Bootstrap, blue) for each taxonomic level.

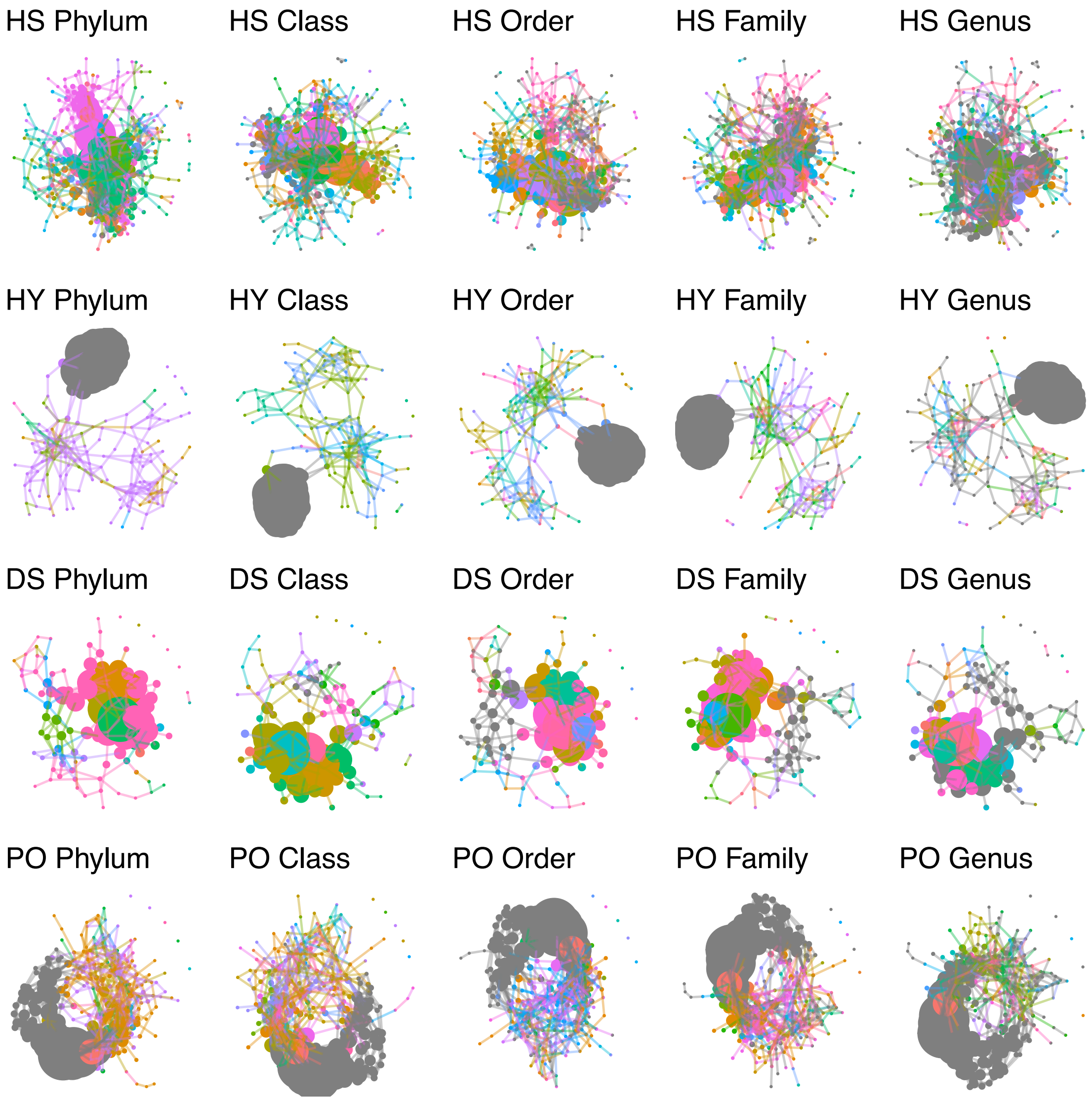

Supplementary Fig. S25. Hub networks at different taxonomic levels and environments. Taxonomic levels are arranged by row and environments are arranged by column. Nodes are sized as function of hub score, using the same scale as in Figure 5. Nodes are colored by genus classification with ambiguous, unassigned or uncultured taxa depicted in dark gray. Within each environment, similar relevance for unknown taxa is observed across taxonomic levels. PO: Polar, DS: Deep sea, HS: Hot springs, HY: Hypersaline.

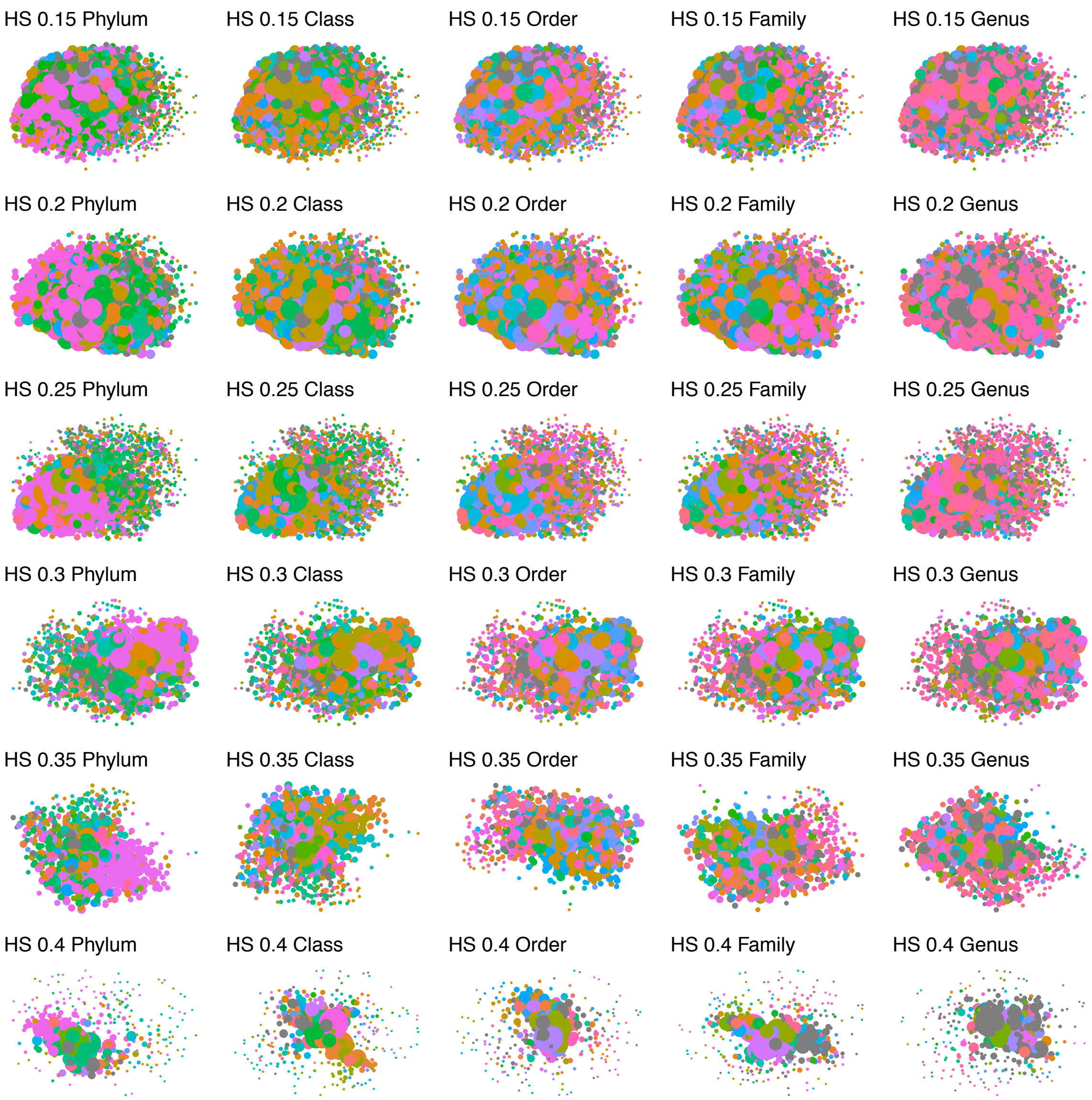

Supplementary Fig. S26. Hub networks using a range of sample percentage thresholds for hot springs habitats. From top to bottom, networks are shown for direct co-occurrence relationships for all hot springs (HS) taxa present in ≥ 15% of all samples to ≥ 40% of all samples in increasing order (i.e., more stringent criteria). Taxonomic levels are arranged by row and prevalence thresholds are arranged by column. Nodes are sized as function of hub score, using the same scale as in Figure 5. Nodes with an unknown designated classification at each taxonomic level are depicted in gray.

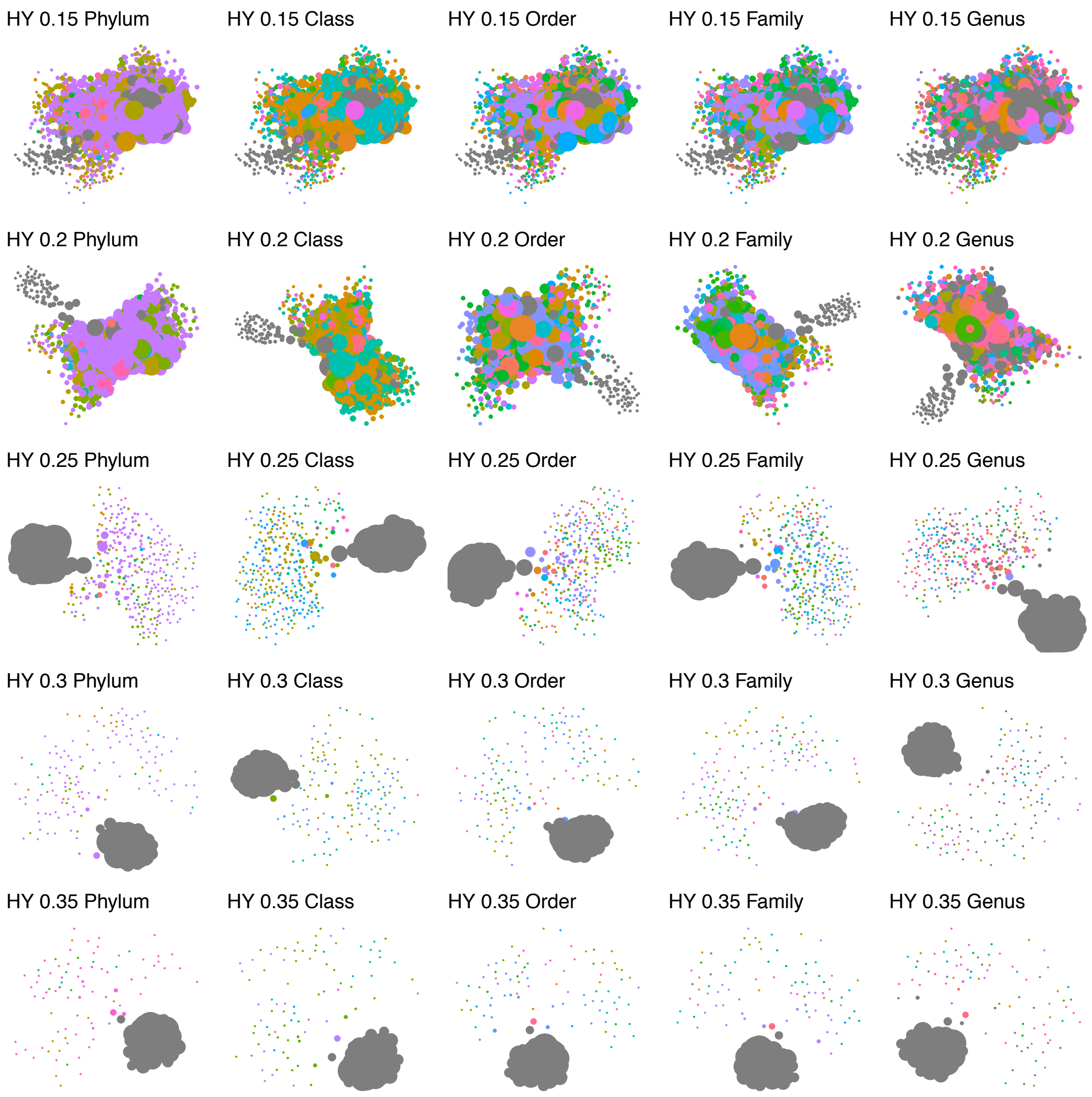

Supplementary Fig. S27. Hub networks using a range of sample percentage thresholds for hypersaline habitats. From top to bottom, networks are shown for direct co-occurrence relationships for all hypersaline (HY) taxa present in ≥ 15% of all samples to ≥ 40% of all samples in increasing order (i.e., more stringent criteria). Taxonomic levels are arranged by row and prevalence thresholds are arranged by column. Nodes are sized as function of hub score, using the same scale as in Figure 5. Nodes with an unknown designated classification at each taxonomic level are depicted in gray.

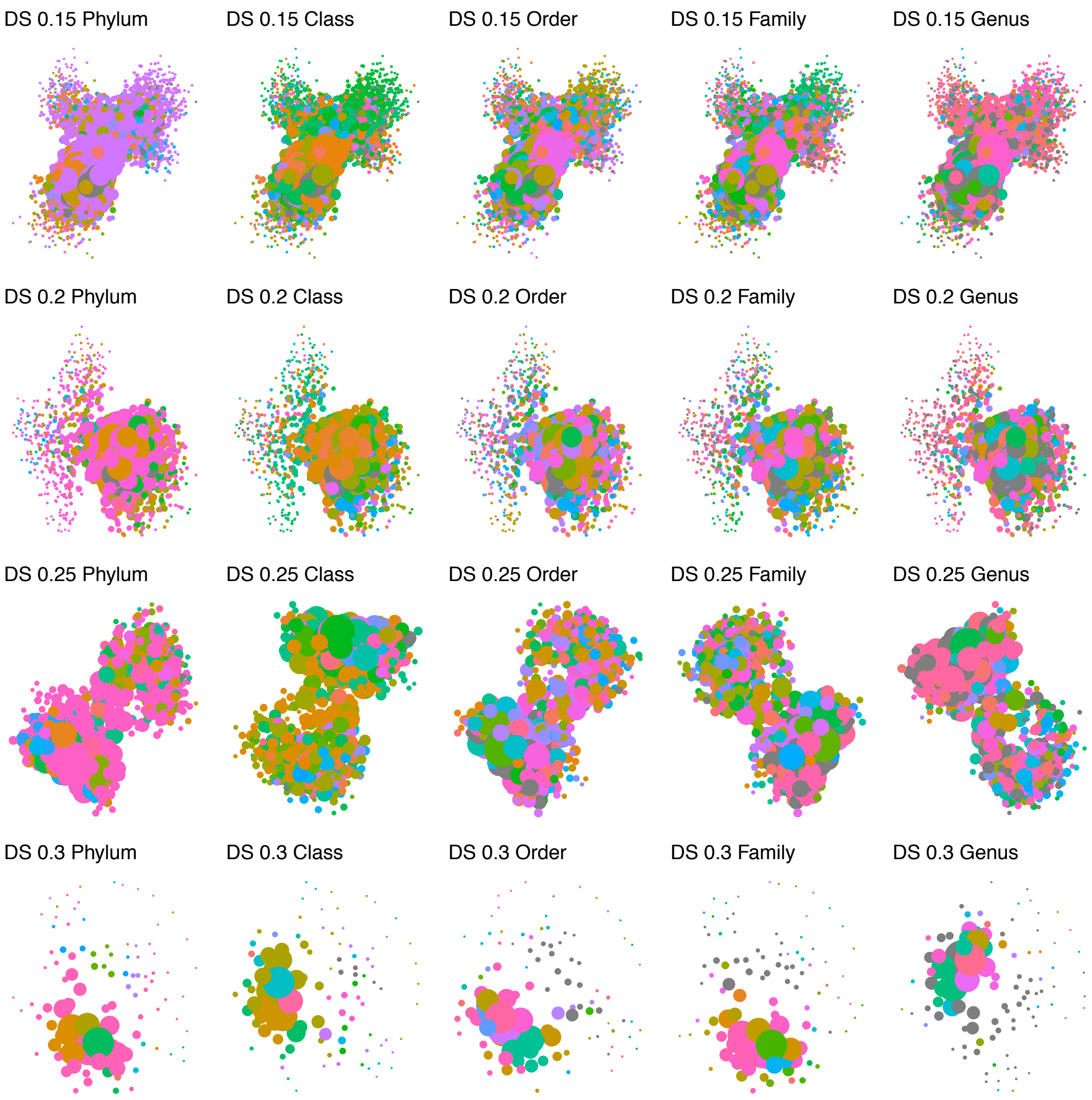

Supplementary Fig. S28. Hub networks using a range of sample percentage thresholds for deep sea habitats. From top to bottom, networks are shown for direct co-occurrence relationships for all deep sea (DS) taxa present in ≥ 15% of all samples to ≥ 40% of all samples in increasing order (i.e., more stringent criteria). Taxonomic levels are arranged by row and prevalence thresholds are arranged by column. Nodes are sized as function of hub score, using the same scale as in Figure 5. Nodes with an unknown designated classification at each taxonomic level are depicted in gray.

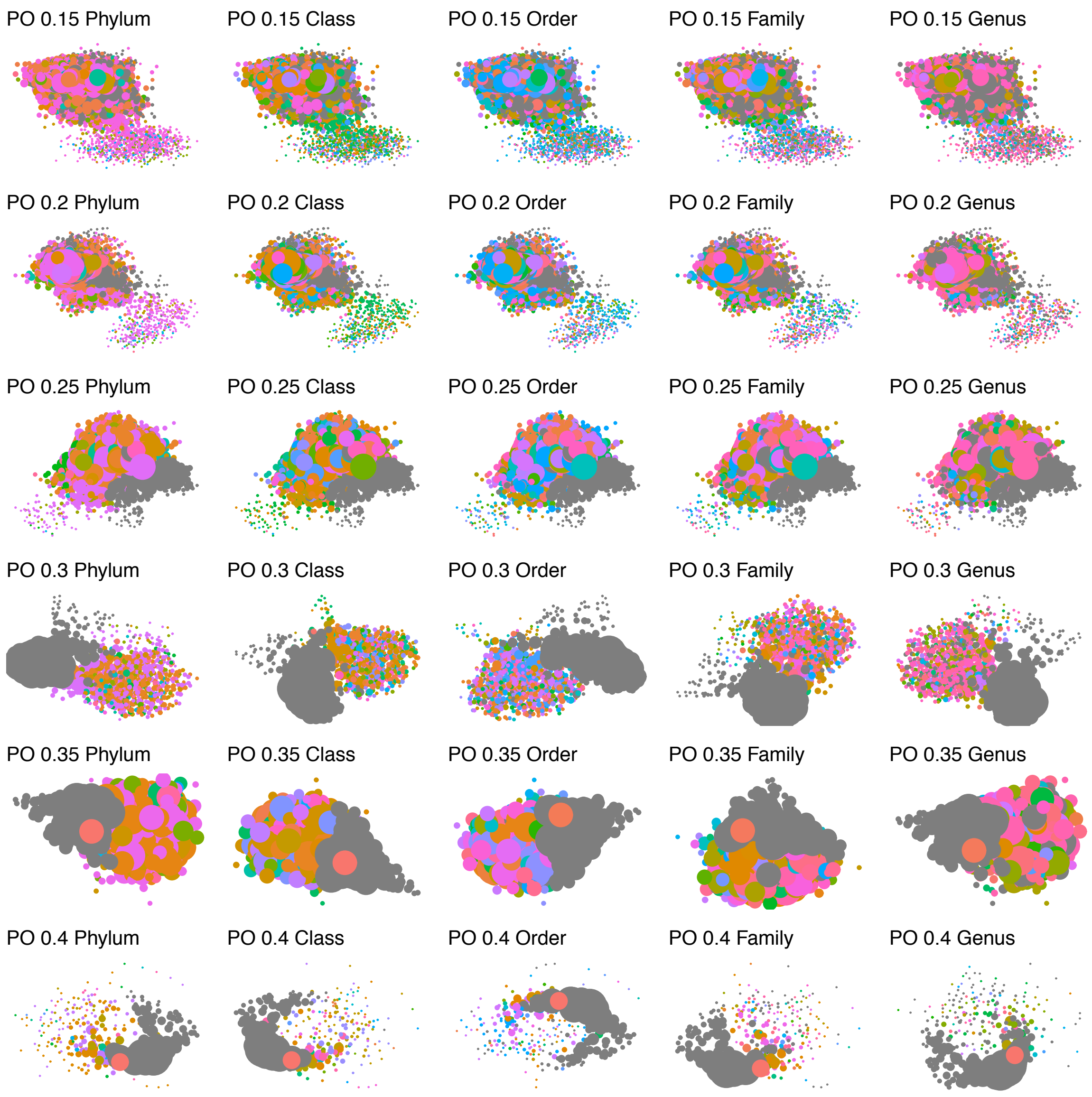

Supplementary Fig. S29. Hub networks using a range of sample percentage thresholds for polar habitats. From top to bottom, networks are shown for direct co-occurrence relationships for all polar (PO) taxa present in ≥ 15% of all samples to ≥ 40% of all samples in increasing order (i.e., more stringent criteria). Taxonomic levels are arranged by row and prevalence thresholds are arranged by column. Nodes are sized as function of hub score, using the same scale as in Figure 5. Nodes with an unknown designated classification at each taxonomic level are depicted in gray.

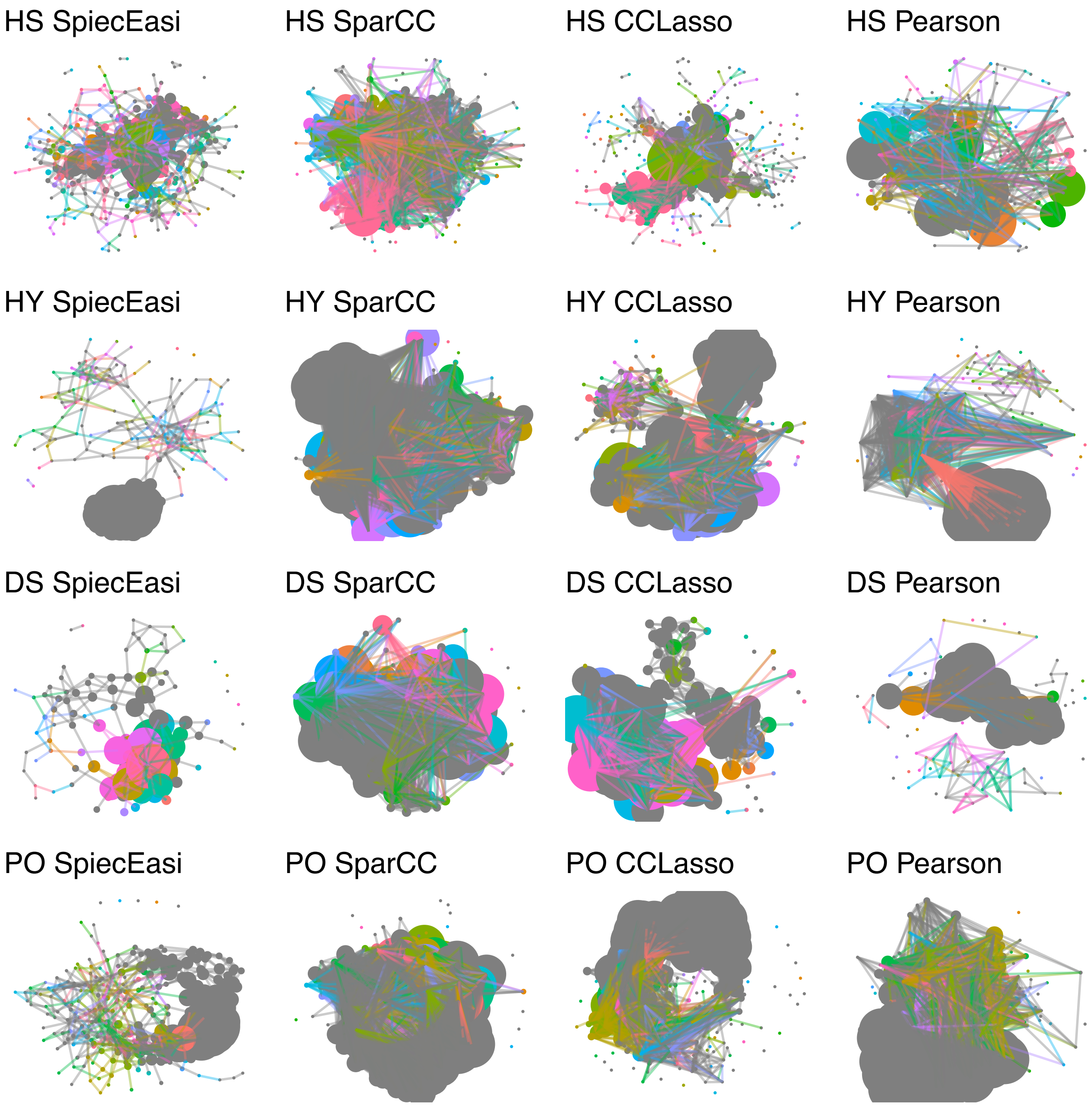

Supplementary Fig. S30. Hub networks at genus-level using different correlation and regression metric tools. From top to bottom, hub networks at genus-level are shown for hot springs (HS), hypersaline (HY), deep sea (DS), and polar (PO) environments, using different network correlation tools. Taxonomic levels are arranged by row and network construction algorithms (SpiecEasi, SparCC, CCLasso, Pearson) are arranged by column. Thresholds for each network construction tool are the same as mentioned in Supplementary Figure S5.

Supplementary Table 1. Key metabolic pathways and extreme environmental stress response genes

Environment	Response mechanism	Key genes
Extremophilic and Poly-extremophilic (1-3)	General stress response and Biofilm formation	*spoT, RpoS*, polyphosphate kinases, ABC transporters, reverse gyrase
	Metal resistance	NiFe and membrane-bound Hydrogenases, iron oxidases, arsenate reductase, *mer* operon, cobalt-zinc-cadmium and copper resistance genes
	Motility and adhesion	*pilM*, flagellar biosynthesis and chemotaxis genes, extracellular polymeric substance (EPS) genes, *gal* operon
	Carbon assimilation and fixation	Phosphoribulokinase (*cbbP*), RuBisCo enzymes, BCT1 transporter for rTCA cycle
	Sulfur oxidation and assimilation	*tetH*, heterosulfide reductase complex, sulfur oxygenase reductase, *soxXYZAB*
	Nitrogen fixation and assimilation	Nitrogenase complex (Nif), nitrate and nitrite reductases, ammonia permease
Thermophilic (4, 5)	Heat shock response	HSP60 and HSP10
	Glycolysis Activation	GAPDH, PGK, PGL
	Cytoplasm-induced hyperthermostability	Intracellular protease, Thioredoxin reductase, hydroperoxide reductase
Psychrophilic (6)	Cold shock response	CSP (cold shock protein) family, DEAD-box protein A
	Cell wall, membrane lipid modifications	LPS glycosyltransferase, PHA synthesis genes
	Fatty acid degradation	Fatty acid desaturase, trehalose synthase
Halophilic and Acidophilic (4)	Osmotic shock response	*yggT, yggS, yggU*
	Ectoine Biosynthesis and sucrose metabolism	*arR, ectA, ectB, ectC, eutB, eutC, eutD, ,ehuA, ehuB, ehuC, ehuD, spp, sps*
	Chloride entry Control	Chloride channel, o*mpA*
	Sodium efflux and potassium transport	*nqrA, trkA, atpC, gdhB, trkH, kpdA, kpdB, kpdC, kpdD, kpdE*, Na+/H+ *mrp* encoded antiporter

Supplementary Table 2. Results of hub blast against 100 metagenomes.

Environment	Hub Type	Total Scaffold Count	Adapt Matches (Total)	Adapt Matches (Avg)	Hyp Gene Count (Total)	Hyp Gene Count (Avg)	Adapt Operon Count (Total)	Adapt Operon Count (Avg)
**Hot Springs**	K	53	185	3.49	1430	27.0	121	2.62
	UNK	81	263	3.29	1832	22.9	172	2.75
**Hypersaline**	K	66	285	4.32	2309	35.0	173	2.28
	UNK	8	34	4.25	248	31.0	22	2.15
**Deep Sea**	K	5	17	3.40	103	20.6	8	1.6
	UNK	5	15	3.00	64	12.8	3	0.6
**Polar**	K	9	22	3.67	158	26.3	13	2.56
	UNK	9	51	5.67	230	25.6	23	2.17
**Total**		236	872		6374		535	

K: Known, UNK: Unknown at genus level
Adapt: Adaptation, Hyp: Hypothetical

Bibliography

1. Orell A, Navarro CA, Rivero M, Aguilar JS, Jerez CA. Inorganic polyphosphates in extremophiles and their possible functions. Extremophiles. 2012;16(4):573-83.
2. Seufferheld MJ, Alvarez HM, Farias ME. Role of polyphosphates in microbial adaptation to extreme environments. Applied and environmental microbiology. 2008;74(19):5867-74.
3. Park C, Park W. Survival and Energy Producing Strategies of Alkane Degraders Under Extreme Conditions and Their Biotechnological Potential. Frontiers in microbiology. 2018;9:1081-.
4. Gunde-Cimerman N, Plemenitaš A, Oren A. Strategies of adaptation of microorganisms of the three domains of life to high salt concentrations. FEMS Microbiology Reviews. 2018;009:353-75.
5. Zeldes BM, Keller MW, Loder AJ, Straub CT, Adams MWW, Kelly RM. Extremely thermophilic microorganisms as metabolic engineering platforms for production of fuels and industrial chemicals. Frontiers in Microbiology. 2015;6:1209-.
6. Tribelli P, López N, Tribelli PM, López NI. Reporting Key Features in Cold-Adapted Bacteria. Life. 2018;8(1):8-.
